# Sensitivity analysis of dynamic biological systems with time-delays

**DOI:** 10.1186/1471-2105-11-S7-S12

**Published:** 2010-10-15

**Authors:** Wu Hsiung Wu, Feng Sheng Wang, Maw Shang Chang

**Affiliations:** 1 Department of Computer Science and Information Engineering, National Chung Cheng University, Chiayi 62102, Taiwan; 2 Department of Chemical Engineering, National Chung Cheng University, Chiayi 62102, Taiwan

## Abstract

**Background:**

Mathematical modeling has been applied to the study and analysis of complex biological systems for a long time. Some processes in biological systems, such as the gene expression and feedback control in signal transduction networks, involve a time delay. These systems are represented as delay differential equation (DDE) models. Numerical sensitivity analysis of a DDE model by the direct method requires the solutions of model and sensitivity equations with time-delays. The major effort is the computation of Jacobian matrix when computing the solution of sensitivity equations. The computation of partial derivatives of complex equations either by the analytic method or by symbolic manipulation is time consuming, inconvenient, and prone to introduce human errors. To address this problem, an automatic approach to obtain the derivatives of complex functions efficiently and accurately is necessary.

**Results:**

We have proposed an efficient algorithm with an adaptive step size control to compute the solution and dynamic sensitivities of biological systems described by ordinal differential equations (ODEs). The adaptive direct-decoupled algorithm is extended to solve the solution and dynamic sensitivities of time-delay systems describing by DDEs. To save the human effort and avoid the human errors in the computation of partial derivatives, an automatic differentiation technique is embedded in the extended algorithm to evaluate the Jacobian matrix. The extended algorithm is implemented and applied to two realistic models with time-delays: the cardiovascular control system and the TNF-*α *signal transduction network. The results show that the extended algorithm is a good tool for dynamic sensitivity analysis on DDE models with less user intervention.

**Conclusions:**

By comparing with direct-coupled methods in theory, the extended algorithm is efficient, accurate, and easy to use for end users without programming background to do dynamic sensitivity analysis on complex biological systems with time-delays.

## Background

Mathematical modeling has been applied to the study and analysis of complex biological systems for a long time. Many mathematical models for dynamic biological systems are formulated as nonlinear ordinary differential equations (ODEs). Some processes in biological systems, such as the gene expression and feedback control in signal transduction networks, involve a time delay. These systems are represented as delay differential equation (DDE) models. Many DDE models have been proposed in the last decade [1-3]. Bocharov et al. [4] reviewed various applications of DDE models in population dynamics, epidemiology, immunology, neural networks, and cell kinetics. Sensitivity analysis can shed light on the dynamic behavior of biological systems and assist the modeling process by identifying the critical parameters that determine the essential behavior of the system. Numerical sensitivity analysis of a DDE model by the direct method requires to obtain the solutions of model and sensitivity equations with time-delays. To do dynamic sensitivity analysis on DDE models, an efficient and accurate approach to compute the solution of DDEs is the basic requirement. Many sophisticated DDE solvers are available recently [5-12]. The major effort is the computation of Jacobian matrix when computing the solution of sensitivity equations. The computation of partial derivatives of complicated equations either by the analytic method or by symbolic manipulation is time consuming, inconvenient, and prone to introduce human errors. To surmount this problem, an automatic approach to obtain the derivatives of complex functions efficiently and accurately is necessary.

Dynamic sensitivity analysis is an important tool for assessing dynamic behavior of biological systems. The common used approach for sensitivity analysis is the numerical differentiation by finite difference approximation. The main drawback of this approach is that the accuracy is hard to analyze. Due to the efficiency and accuracy, a variety of direct methods are proposed [13-15]. Rihan [16] derives a general theory for sensitivity analysis of DDE models by using adjoint equations and direct methods to estimate the sensitivity equations with variable and constant parameters, respectively. The kinetic preprocessor (KPP) numerical library is a comprehensive set of software tools for direct and adjoint sensitivity analysis [17]. An-other approach which can be used to evaluate sensitivity equations is automatic differentiation. Automatic differentiation is a non-approximate differentiation technique that permits the fast and accurate evaluation of partial derivatives in Jacobian matrix. The values for the derivatives obtained by automatic differentiation are exact if we do not take account of the unavoidable round-off error due to the finite precision arithmetic of a computer. The theoretical exactness of the automatic differentiation comes from the fact that it uses the same rules of differentiation as in differential calculus, but these rules are applied to an algorithmic evaluation of the function rather than to a formula. The implementation of automatic differentiation can be divided into two different classes: source code transformation and operator overloading. The most widely used source code transformation program is ADIFOR 2.0 [18]. This program, like as the preprocessor of a compiler, accepts and analyzes Fortran 77 source code and produces code to evaluate the derivatives of the function in Fortran 77 standard. The output code is optimized by eliminating unnecessary arithmetic operations and temporary variables and then compiled with a standard compiler into an object code that can simultaneously evaluate derivatives and function values. Hwang et al. [19] demonstrated that ADIFOR is a powerful tool for ODE models from three aspects of performance: accuracy, efficiency, and scaled capability. Griewank et al. [20] developed an open-source code, automatic differentiation by overloading in C++ (ADOL-C), for the automatic differentiation of C and C++ programs. The implementation of ADOL-C utilizes the operator overloading capability of C/C++ compilers that accept user-defined data types, operators and functions. The implementation of either the source code transformation or the operator overloading is a compile-time solution. It allows one to generate derivatives from complicated existing code or user-provided model equations that expressed by user-defined data types, operators and functions. These available codes are implemented for ODEs and is suitable for users with programming background.

We have proposed an efficient algorithm with an adaptive step size control, called adaptive modified collocation method (AMCM), to compute the solution and dynamic sensitivities of biological systems described by ODEs [21]. The algorithm is extended to solve the solution and dynamic sensitivities of time-delay systems described by DDEs in this paper and named as extended adaptive modified collocation method (EAMCM). The EAMCM is implemented as a user-friendly program that facilitates the dynamic sensitivity analysis of DDE models through the implementation of adaptive direct-decoupled method and automatic differentiation. EAMCM requires the user to supply only the model equations at run-time in a form of mathematical expression to compute dynamic sensitivities of DDE models. The evaluation of sensitivity equations is done automatically by automatic differentiation technique along with the inevitable evaluation of model equations. By combining the adaptive direct-decoupled AMCM algorithm with automatic differentiation technique and extending its usage to DDE models, the extended algorithm, EAMCM, is efficient, accurate, and easy to use for end users without programming background to do dynamic sensitivity analysis on complex biological systems with time-delays.

To evaluate the applicability of the extended algorithm, it is applied to two realistic models with time-delays: the cardiovascular control system and the TNF-*α *signal transduction network. The first DDE model for human cardiovascular control system was developed by Fowler et al. [22] to explore the interactions between the heart rate and blood pressure under the baroreflex control. The time delay arises from the slow activity of sympathetic nervous system. Sensitivity analysis is applied to this DDE model through the EAMCM program to identify the key parameters that could provide useful diagnostic information about the state of the cardiovascular system. The second DDE model for TNF-*α *signal transduction network built by Rangamani and Sirovich [23] considers both the NF-*κ*B-mediated survival pathway and the caspase-mediated apoptosis pathway simultaneously. Due to the delay of translocation of NF-*κ*B to the nucleus, the transcription processes of cIAP and I*κ*B in the NF-*κ*B-mediated survival pathway were represented by DDEs. The EAMCM is applied to this model to analyze its dynamic sensitivities and decipher the relationship between the NF-*κ*B-mediated survival pathway and the caspase-mediated apoptosis pathway.

## Results and discussion

Cardiovascular disease is the major cause of human death. A detailed understanding of cardiovascular systems is important for the diagnosis of cardiovascular disease, ultimately leading to improved treatment. The EAMCM program can be used to do dynamic sensitivity analysis on the cardiovascular control system to investigate the hemodynamics and regulation control of cardiovascular systems.

Apoptosis is central to the development of cancer. In the recent years, the prevalent model to explain why cancer therapies fail has been that cell resistant to or inhibition of apoptosis [24]. So now, the new treatment goal is how to control apoptosis that brings on cell death of the cancer cells. NF-*κ*B is a transcription factor family that activating numerous genes that are related to cell survival pathways. Most commonly, NF-*κ*B activation inhibits apoptosis pathways, as evidenced by several knockout mouse models [25,26]. Based on these findings, the goal to design more effective cancer therapies can be initiated by apoptosis induction and inhibition of NF-*κ*B. Many mathematical models describing the dynamics of apoptosis and NF-*κ*B pathways have been published in last decade [27-31]. Most of the models to date have concentrated on only one of signaling pathways and do not consider the delayed transcription processes. The EAMCM program is applied to a TNF-*α*-induced signaling network considering both signaling pathways simultaneously to investigate how these two pathways work together to regulate cell fate in response to TNF-*α*.

### Cardiovascular control system

Mathematical models are useful to investigate the hemodynamics and regulation control of cardiovascular systems. In general, cardiovascular models consist of two major systems: the hemodynamic system and the autonomic nerve control system. The hemodynamic system is a systemic circulatory blood distribution network to deliver oxygen, nutrients, and hormones to cells and remove carbon dioxide and metabolic wastes. The left ventricle pumps blood to arteries, capillaries, veins, and back to the heart. The blood hemodynamics of this circulation can be represented by the relationship between blood pressure and heart rate in the cardiovascular system. The control of the blood pressure is crucial to human health due to that the blood pressure is the energy, generated by the heart, to push blood around the body. Although the endogenous regulation of arterial pressure is not completely understood, there are evidences that baroreceptors are important for minimizing changes in blood pressure. Animal studies have shown that blood pressure is much more variable if the influence of baroreceptors is removed [32,33]. Baroreceptors detect changes in arterial pressure and send signals ultimately to the medulla of the brain stem. The medulla, by way of the autonomic nerve control system, adjusts the mean arterial pressure by altering the heart rate and the total peripheral resistance. The autonomic nerve control system includes the sympathetic and parasympathetic nervous systems. When blood pressure starting to fall, the baroreceptor stimulation decreasing and the reflex response causes the peripheral resistance increasing and the heart to beat faster and harder by slow-acting sympathetic nerves. This negative-feedback mechanism largely restores the blood pressure. Conversely, if blood pressure increases, stimulation of baroreceptors gives rise to nerve impulses which run to the brain and slow down the heartbeat through the fast activity in the parasympathetic pathway.

Fowler et al. [22] developed a lumped DDE model of the integrated cardiovascular system combined with a baroreflex feedback control of blood pressure to describe the interactions between heart rate, blood pressure, and respiration. This DDE model is a simple model without considering the pulmonary part of the cardiovascular system and is derived from the model introduced by Ottesen [34] by adding an intrinsically controlled heart rate and baroreflex control of peripheral resistance. This simple model consists of only 2 delay differential equations which include 16 parameters and is expressed as

(1) $$\frac{\text{d}x_{1}}{\text{d}t^{*}}=\frac{h_{0}}{\varepsilon_{h}}\left[\frac{\beta g_{1}}{1+\gamma g_{2}}-\nu g_{2}\right]+\frac{\delta }{\varepsilon_{h}}(h_{0}-x_{1}),$$

(2) $$\frac{\text{d}x_{2}}{\text{d}t^{*}}=\frac{\mu p_{0}}{\varepsilon_{p}h_{0}}x_{1}-\frac{x_{2}}{\varepsilon_{p}(1+\alpha g_{1})},$$

where *x*_1 _is the heart rate, *x*_2 _is the arterial pressure, and t* = *t*/*τ *is the dimensionless time. The functions *g*_1 _and *g*_2 _are defined by

$$\begin{array}{lll} g_{1} & = & \frac{1}{1+{\left[x_{2}(t^{*}-1)/p_{0}+r_{1}\right]}^{n}},\\ g_{2} & = & 1-\frac{1}{1+{\left[x_{2}(t^{*})/p_{0}+r_{2}\right]}^{n}},\\ r_{1} & = & A_{1}\sin\{2\pi f_{r}\tau (t^{*}-1)-\phi \},\\ r_{2} & = & A_{2}\sin\{2\pi f_{r}\tau t^{*}-\phi \}, \end{array}$$

where *x*_2_(t* - 1) and *x*_2 _(t*) denote the blood pressure with and without a time delay, respectively. The values and definitions of system parameters are given in Table 1. The state variables are the heart rate and blood pressure. A sinusoidal function is added to the model equations to mimic respiration. The Hill function *g_i _*with an exponent *n *is used to model the baroreflex feedback control of heart rate. This simple model has shown to be able to reproduce many of the empirical observations such as respiratory sinus arrhythmia (RSA), Mayer waves, and synchronization [35].

**Table 1:** The value and definition of system parameters

Parameter	Definition	Value
*h*_0_	Uncontrolled heart rate	100 bpm
*p*_0_	Mean arterial blood pressure	100 mm Hg
*α*	Sympathetic effect on peripheral resistance	15
*β*	Sympathetic control of heart rate	10
*ν*	Strength of vagal tone	9.63
*δ*	Relaxation time	0.8 s^-1^
*γ*	Damping effect of vagal activity on the sympathetic tone	0.2
*μ*	3/(2 + *α*)	0.18
*A*_1_	Amplitude of the influence of respiration on blood pressure	0
*A*_2_	Amplitude of the influence of respiration on heart rate	0.003
*f*_r_	Breathing rate	0.17 Hz
*τ*	Sympathetic time delay	3 s
*ϕ*	Phase lag	3.14 s
*n*	Hill exponent	8
*ε_h_*	Relative coefficient for heart rate	1
*ε_p_*	Relative coefficient for blood pressure	3

The definition of system parameters in the DDE model developed by Ottesen [34]. The parameter values are obtained from the literature [35].

**Figure 1:**
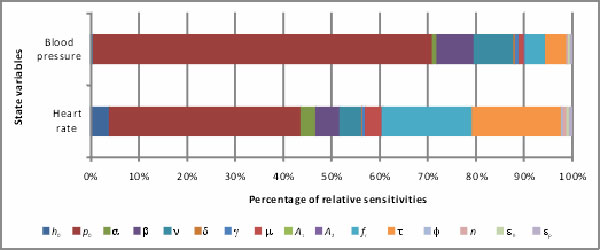
**Stacked 100% column chart for individual state variables**. Each column in the stack column chart shows all relative parameter sensitivities for a state variable. The proportion of a parameter sensitivity to the total sensitivity for a state variable is displayed as a color area in each column. The values of time-averaged relative parameter sensitivities are used as the data.

**Figure 2:**
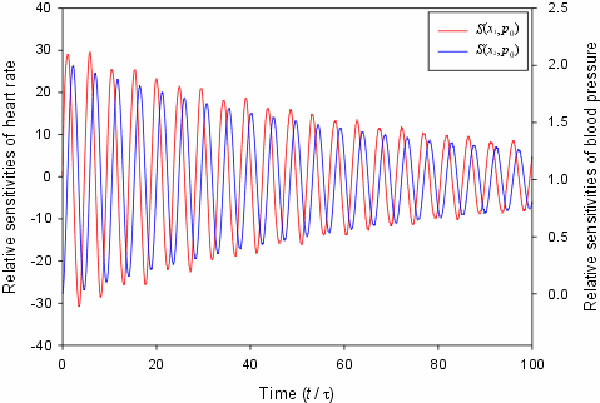
**The relative sensitivities of heart rate and blood pressure with respect to *p*_0_**. The relative sensitivities of heart rate and blood pressure with respect to the uncontrolled average arterial blood pressure. The time is in dimensionless scale.

**Figure 3:**
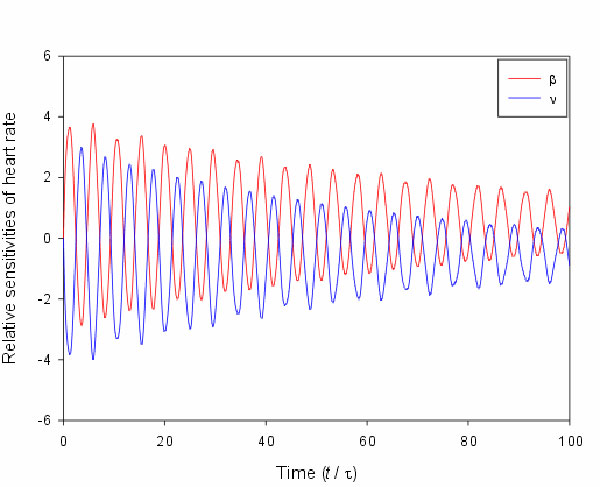
**The relative sensitivities of heart rate with respect to *β *and *ν***. The relative sensitivities of heart rate with respect to parameters for slow sympathetic control and fast vagal control. The time is in dimensionless scale.

**Figure 4:**
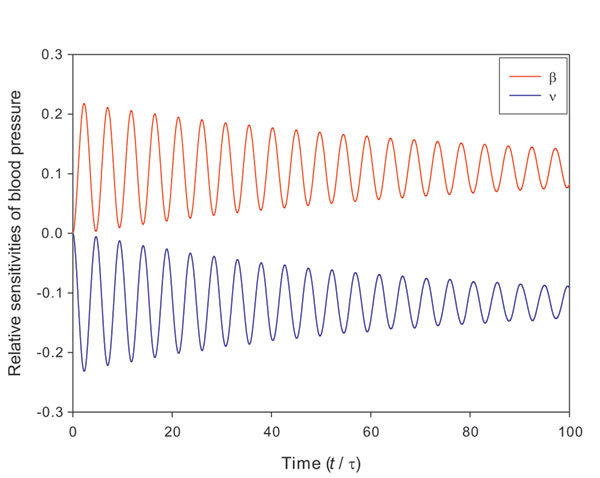
**The relative sensitivities of blood pressure with respect to *β *and *ν***. The relative sensitivities of blood pressure with respect to parameters for slow sympathetic control and fast vagal control. The time is in dimensionless scale.

The EAMCM program is used to do sensitivity analysis on the lumped cardiovascular model. The non-constant exponent of Hill function and sinusoidal functions in differential model equations complicate the evaluation of Jacobian matrix for computing the solution of sensitivity equations. By the help of automatic differentiation embedded in the EAMCM program, user can provide the model equations only at run-time for solving the dynamic sensitivities of the cardiovascular system. The dynamic sensitivities of heart rate and blood pressure with respect to all system parameters and initial conditions are computed. All relative parameter sensitivities are presented by 100% stacked column chart and shown in Figure 1. It is easy to find which parameter makes more effects on heart rate and blood pressure than the others from Figure 1. The values of top five sensitivities for the heart rate and blood pressure are shown in Table 2. The uncontrolled average arterial blood pressure (*p*_0_), breathing rate (*f*_*r*_), sympathetic delay (*τ*), sympathetic control of heart rate (*β*), and strength of vagal tone (*ν*) are identified as the key parameters for the control of heart rate and blood pressure. The relative sensitivities of heart rate and blood pressure with respect to the uncontrolled average arterial blood pressure are shown in Figure 2. The dynamic sensitivities of heart rate with respect to *p*_0 _oscillate symmetrically between positive and negative values. This result indicates that the uncontrolled average arterial blood pressure amplifies the variation of heart rate. In contrast, the dynamic sensitivities of blood pressure with respect to *p*_0 _oscillate but are all positive. This means that an increase of the uncontrolled average arterial blood pressure shifts the blood pressure to a higher value but does not change the variation of blood pressure. As shown in Table 2, the effect of uncontrolled average arterial blood pressure on the variation of average heart rate is tenfold larger than the variation of blood pressure. There is evidence that the slow-acting sympathetic nerves and the fast-acting vagal nerves compete with each other to increase and decrease the heart rate, respectively [36]. The relative sensitivities of heart rate with respect to parameters for slow sympathetic control (*β*) and fast vagal control (*ν*) are investigated and shown in Figure 3. Figure 3 shows the sympathovagal balance in physiology and both sympathetic control and vagal control amplify the variation of heart rate. The relative sensitivities of blood pressure with respect to parameters for slow sympathetic control (*β*) and fast vagal control (*ν*) are shown in Figure 4. The slow-acting sympathetic control upregulates the blood pressure, but does not change its variation. The relative sensitivity of blood pressure with respect to the sympathetic control is positive over the time. In contrast, the fast-acting vagal control downregulates the blood pressure and has a negative relative sensitivity over the time.

**Table 2:** The ranking of relative sensitivities of heart rate and blood pressure

Rank	1	2	3	4	5
Heart rate	*p*_0_	*f_r_*	*τ*	*β*	*v*
	10.667	4.791	4.791	1.284	1.237
	39.97%	18.61%	18.61%	4.81%	4.63%

Blood pressure	*p*_0_	*v*	*β*	*f_r_*	*τ*
	1.000	0.116	0.109	0.061	0.061
	70.56%	8.18%	7.68%	4.30%	4.30%

The dynamic sensitivities of heart rate (*h*) and blood pressure (*p*) with respect to all parameters are ranked based on the time-averaged relative sensitivities over the response time. The values of time-averaged relative sensitivities are shown in the second row and the corresponding ratios to the total relative sensitivity are shown in third row.

### Apoptosis signal network

**Figure 5:**
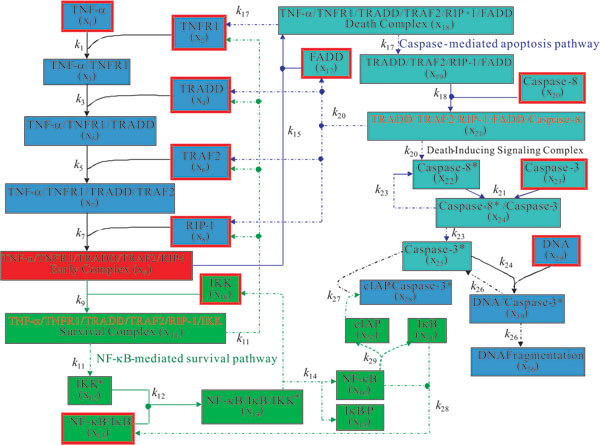
**Schematic diagram of TNF-*α *signal transduction network**. The solid lines indicate reversible reactions and the dash-dot lines denote irreversible reactions. The dash lines indicate the delayed transcription processes. The reactions and components of the survival pathway are shown in green. The reactions and components of the apoptotic pathway are shown in blue. The boxes with red border denote the components with nonzero initial value in the network.

The TNF-*α *signal transduction network was developed by Rangamani and Sirovich [23], which considers both the NF-*κ*B-mediated survival pathway and the caspase-mediated apoptosis pathway simultaneously. These two pathways involve 31 species in 19 reactions and the schematic diagram is shown in Figure 5. The formation of this network involves binding reactions between ligand and death receptor, protein-protein reactions, enzymatic reactions, translocations, and transcription processes. The network is induced by ligation of TNF-*α *to the cell surface receptor TNFR1. The ligation of TNFR1 by TNF results in the recruitment of the adaptor proteins such as TNFR-associated death domain (TRADD), TNFR-associated factor 2 (TRAF2), receptor-interacting protein 1 (RIP1), and possibly other yet unidentified proteins to form the early complex. In the NF-*κ*B-mediated transcription pathway, the inactive inhibitor kappa B kinase (IKK) binds to the early complex leads to the activation of IKK, I*κ*B phosphorylation, and release of NF-*κ*B. The free NF-*κ*B translocates to the nucleus, binds to DNA, and leads to the transcription of I*κ*B and cellular inhibitor of apoptosis protein (cIAP) that protects cells from TNF-induced apoptosis by binding to activated caspase-3 [37]. The newly synthesized free I*κ*B enters the nucleus and binds to nuclear NF-*κ*B and this complex is exported to the cytoplasm [38]. This NF-*κ*B-I*κ*B complex is the target for I*κ*B phosphorylation by active IKK and the liberating NF-*κ*B will translocate to the nucleus again. Nelson et al. [39] proposed that this oscillatory feedback behavior of NF-*κ*B regulates the expression of cIAP. In the caspase-mediated apoptosis pathway, TRADD, RIP1, and TRAF2 are dissociated from TNFR1 and recruit Fas Associated Death Domain (FADD) and caspase-8 to form a protein complex called the death-inducing signaling complex (DISC) [40]. As a result of DISC formation, caspase-8 is cleaved at the DISC resulting in the activation of caspase-8. The activated caspase-8 in turn activates effector caspases, such as caspase-3, causing the cell to undergo apoptosis.

The transcription processes of cIAP and I*κ*B due to the translocation of NF-*κ*B to the nucleus are represented as delayed reactions. The delay time used for transcription is 20 minutes as suggested by Sung and Simon [41]. Based on material balance, this model consists of 31 delay differential equations which include 29 parameters. The state variables are the concentration of the molecules in the survival and apoptosis pathways and the input variable is the concentration of TNF-*α *that stimulates the signal transduction pathways. The output variable is the concentration of fragmented DNA, which can be used as a marker for apoptosis. The fragmented DNA is defined as the fraction of DNA sites that have been attacked by the activity of effector caspase. The set of delay differential equations, all of the relevant definitions of variables, and parameters appearing in the DDE model, together with the nominal values can be found in Additional file 1. The reason for representing the model equations here and not just referring to the article by Rangamani et al. [23] is that some of the parameters and state variables have different names as that in the original model.

**Figure 6:**
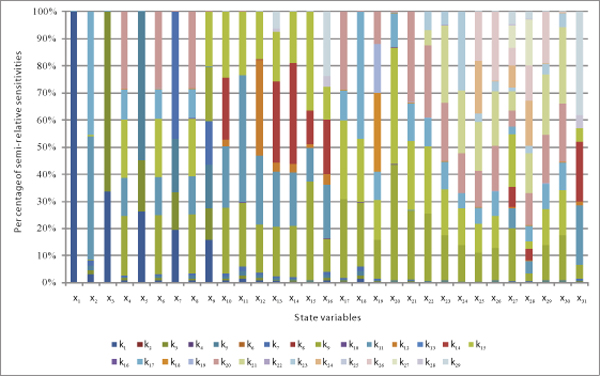
**Stacked 100% column chart for individual state variables**. Each column in the stack column chart shows all semi-relative parameter sensitivities for a state variable. The proportion of a parameter sensitivity to the total sensitivity for a state variable is displayed as a color area in each column. The values of time-averaged semi-relative parameter sensitivities are used as the data.

The EAMCM program is applied to the TNF-*α *signal transduction model using the initial conditions as described in Table S1 of Additional file 1. All dynamic sensitivities with respect to 29 parameters and 31 initial conditions are computed simultaneously without any difficulty. All time-averaged semi-relative parameter sensitivities for each state variable are shown in Figure 6. Most of the time-averaged semi-relative parameter sensitivities for each state variable are too small compared with the largest and can be ignored. It is easy to find from Figure 6 that only some few parameter sensitivities get significant percentage of the total sensitivity for each state variable.

The dynamic sensitivity profiles for all species with respect to *k*_9_, the rate constant of the formation of survival complex, are nearly identical to that with respect to *x*_10_(0), the initial value of IKK (data not shown here). This is not surprising because the kinetic order is set to one for each flux in the model. So each relative effect on the output with respect to the rate constant is the same as that with respect to the initial concentration of the corresponding species. The same situation can be found for each pair of *k*_15 _/*x*_17_(0), *k*_18_/*x*_20_(0), etc. In the following, we analyze the dynamic parameter sensitivities only, because the same results for the corresponding dynamic initial sensitivities can be found from the dynamic parameter sensitivities.

**Figure 7:**
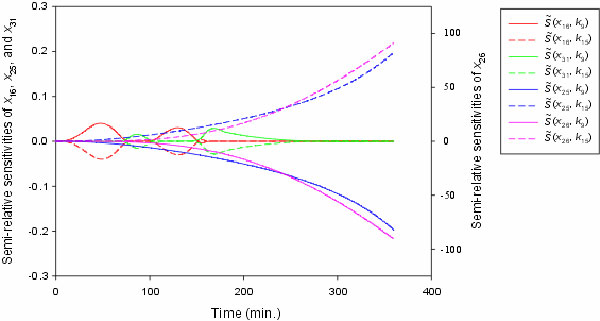
**The symmetry of semi-relative sensitivities with respect to *k*_9 _and *k_15_***. The solid lines are the semi-relative sensitivities with respect to the rate constant *k*_9 _of the formation of survival complex and the short dash lines are the semi-relative sensitivities with respect to the rate constant *k*_15 _of the formation of death complex. The semi-relative sensitivities of NF-*κ*B (*x*_16_) are shown in red, I*κ*B (*x*_31_) in green, activated caspase-3 (*x*_25_) in blue, and fragmented DNA (*x*_26_) in pink.

All of the dynamic sensitivities with respect to *k*_9_, the rate constant of the formation of survival complex, and *k*_15 _, the rate constant of the formation of death complex, are symmetric with respect to the time axis. This means that if we have plotted the sensitivity profile of a species with respect to *k*_9_, the corresponding sensitivity profile with respect to *k*_15 _can be obtained simply by reflecting about the time axis. To elucidate the effects of IKK (*x*_10_) and FADD (*x*_17_) on the oscillatory behavior of NF-*κ*B (*x*_16_) and I*κ*B (*x*_31_) in the survival pathway, the semi-relative sensitivities of NF-*κ*B and I*κ*B with respect to the rate constants *k*_9 _and *k*_15 _, of the formation of survival complex and death complex are shown in Figure 7. We observe, from Figure 7, the negative regulation of oscillatory behavior of NF-*κ*B and I*κ*B when the rate constant (*k*_15_) or the initial concentration of FADD increases. The reverse effect is seen by increasing the rate constant (*k*_9_) or the initial concentration of IKK. We also investigate the responses of the apoptosis pathway to the variances of FADD and IKK. The activation of effector caspase-3 (*x*_23_) is the finial reaction of the apoptosis pathway, so the concentration of the active caspase-3 (*x*_25_) can be used as the response of the apoptosis pathway. The semi-relative sensitivities of active caspase-3 with respect to *k*_9 _and *k*_15 _are shown in Figure 7. The negative values of the semi-relative sensitivities of active caspase-3 with respect to *k*_9 _show that the active caspase-3 decreases when the rate constant (*k*_9_) or the initial concentration of IKK increases. By contrast, increasing the rate constant (*k*_15_) or the initial concentration of FADD induces the increase of the active caspase-3. These results are in agreement with observations by mutant studies [23] and show the interplay of the apoptotic and survival pathways in response to the variations of IKK and FADD. As Figure 7 shows, a combination of increasing the initial concentration of FADD and decreasing the initial concentration of IKK gets more effects on the DNA fragmentation.

**Figure 8:**
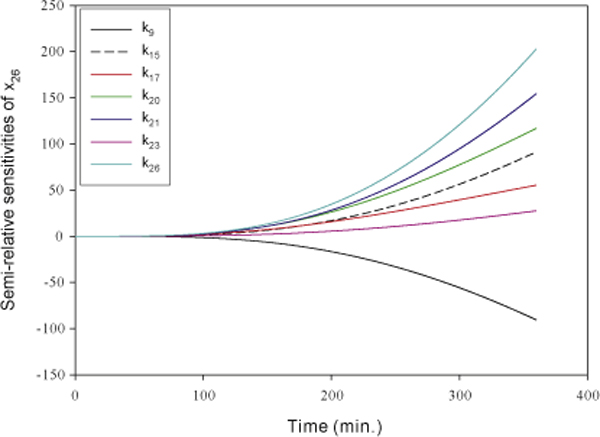
**The semi-relative sensitivities of fragmented DNA**. The semi-relative sensitivities of fragmented DNA (*x*_26_) with respect to the rate constants of the formation of survival complex (*k*_9_), the formation of death complex (*k*_15_), the formation of DISC without TNFR1 (*k*_17_), the caspase-8 activation (*k*_20_), the cleavage of procaspase-3 (*k*_21_), the caspase-3 activation (*k*_23_), and the fragmentation of DNA (*k*_26_).

The fragmented DNA (*x*_26_) is the output of the signal transduction model in response to TNF-*α *stimulus. Our goal is to identify the essential reactions that have significant effect on the system output. The semi-relative sensitivities of fragmented DNA are used to achieve this goal. Since dynamic sensitivities vary with time, it is hard to determine the most important reaction that has the largest effect on the system output. We consider the usual used significance measure, time-averaged semi-relative sensitivity defined similar to equation (12), as the strength of effects on fragmented DNA for individual reactions. Table 3 shows the ranking of dynamic sensitivities of fragmented DNA with respect to all parameters based on time-averaged semi-relative sensitivities. The values of time-averaged semi-relative sensitivities and the corresponding ratios to the total semi-relative sensitivity are shown if the ratios are greater than 2%. Six out of the top seven key parameters belong to the apoptosis pathway. This means that the apoptosis pathway dominates the cell fate in response to TNF-*α*. The rate constants for the formation of phosphorylated caspase-8 (*k*_20_) and phosphorylated caspase-3 (*k*_23_) and the regulation of activating caspase-3 by phosphorylated caspase-8 (*k*_21 _) are identified as important parameters. This result indicates that the activation cascades of caspase-8 and caspase-3 are important reactions in the apoptosis signal transduction pathway. Figure 8 shows the top seven semi-relative sensitivities of fragmented DNA. All key parameters positively regulate the activity of DNA fragmentation except parameter *k*_9_.

**Table 3:** The ranking of semi-relative sensitivities for fragmented DNA

Rank	Parameter	Time-averaged semi-relative sensitivity	Percentage (%)
1	*k*_26_	51.349	25.49
2	*k*_21_	40.336	20.02
3	*k*_20_	33.558	16.66
4	*k*_15_	24.010	11.92
5	*k*_9_	23.755	11.79
6	*k*_17_	17.756	8.81
7	*k*_23_	7.492	3.72

The dynamic sensitivities of fragmented DNA (*x*_26_) with respect to all parameters are ranked based on the time-averaged semi-relative sensitivities. The values of time-averaged semi-relative sensitivities and the corresponding ratios to the total semi-relative sensitivity are shown if the ratios are greater than 2%.

**Figure 9:**
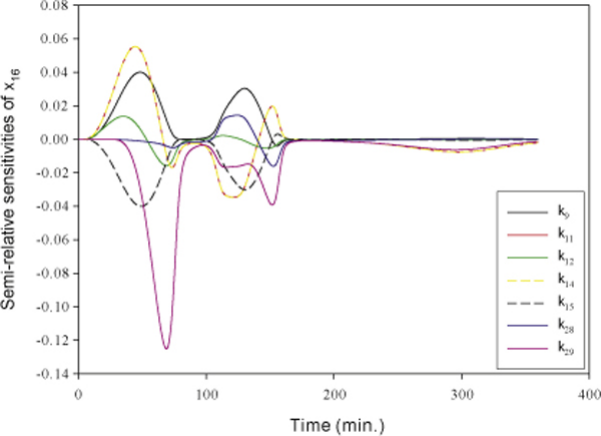
**The semi-relative sensitivities of NF-*κ*B**. The semi-relative sensitivities of NF-*κ*B (*x*_16_) with respect to the rate constants of the formation of survival complex (*k*_9_), the IKK activation (*k*_11_), the formation of NF-*κ*B/I*κ*B/IKK* (*k*_12_), the NF-*κ*B activation (*k*_14_), the formation of death complex (*k*_15_), the deactivation of NF-*κ*B (*k*_28_), and the transcription of cIAP and I*κ*B (*k*_29_).

The transcription factor NF-*κ*B (*x*_16_) plays an important role in the survival pathway. To further understand the control of NF-*κ*B, we analyze the semi-relative sensitivities of NF-*κ*B with respect to all parameters. The ranking of parameter sensitivities of NF-*κ*B based on the time-averaged semi-relative sensitivities is shown in Table 4. A parameter is referred to as an important parameter if the ratio of its time-averaged semi-relative parameter sensitivity to the total parameter sensitivity of NF-*κ*B is greater than 2%. Ignoring the rate constants for the reverse reactions, all parameters -*k*_9_, *k*_11_, *k*_12_, *k*_14_, *k*_28_, and *k*_29 _- in the survival pathway are identified as important parameters. The parameter *k*_15 _is also identified as an important parameter, although NF-*κ*B is negatively sensitive to it. The top seven sensitivities of NF-*κ*B are shown in Figure 9. The dynamic sensitivity of NF-*κ*B with respect to the IKK activation (*k*_11_) is similar to that with respect to the NF-*κ*B activation (*k*_14_) as Figure 9 shows. This means that the inhibitor of IKK acts the same function as the inhibitor of NF-*κ*B to inhibit the activity of NF-*κ*B. The activation of NF-*κ*B in most types of cells leads to the inhibition of apoptosis, accelerates cell proliferation, and promotes tumorigenesis. To inhibit cell growth, some small-molecule inhibitors targeting IKK have already been developed to treat certain type tumors [42]. Several synthetic drugs that are be able to inhibit the activities of IKK and NF-*κ*B have been shown to have the same effects on tumor development [43,44]. Larger the rate constant for the transcription of I*κ*B (*k*_29_), more I*κ*B is generated to deactivate NF-*κ*B and causes lower active NF-*κ*B. A large rate constant for the formation of death complex (*k*_15_) leads to less signals to activate IKK that then phosphorylates I*κ*B and triggers the activation of NF-*κ*B. Both rate constants regulate negatively the activation of NF-*κ*B as shown in Figure 9. The sensitivities of NF-*κ*B with respect to the rates of IKK activation (*k*_11_), the formation of NF-*κ*B/I*κ*B/IKK* (*k*_12_), the NF-*κ*B activation (*k*_14_), and the deactivation of NF-*κ*B (*k*_28_) alternate between positive and negative values. These oscillations of sensitivities may be caused by the negative feedback regulation of I*κ*B.

**Table 4:** The ranking of semi-relative sensitivities of NF-*κ*B

Rank	Parameter	Time-averaged semi-relative sensitivity	Percentage (%)
1	*k*_29_	1.300E-02	23.77
2	*k*_14_	1.082E-02	19.79
3	*k*_11_	1.081E-02	19.76
4	*k*_9_	6.709E-03	12.27
5	*k*_15_	6.672E-03	12.20
6	*k*_12_	2.255E-03	4.12
7	*k*_28_	2.123E-03	3.88

The dynamic sensitivities of NF-*κ*B (*x*_16 _) with respect to all parameters are ranked based on the time-averaged semi-relative sensitivities. The values of time-averaged semi-relative sensitivities and the corresponding ratios to the total semi-relative sensitivity are shown if the ratios are greater than 2%.

**Figure 10:**
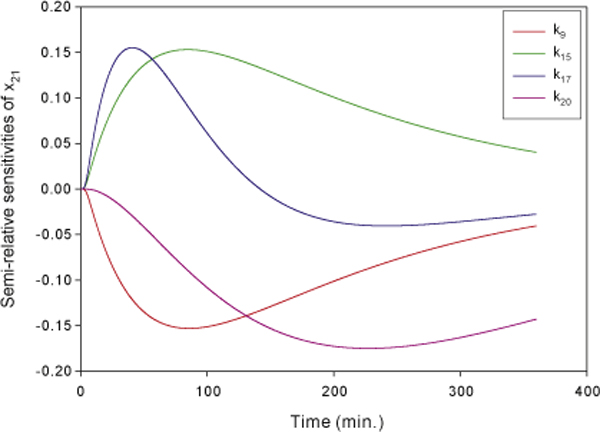
**The semi-relative sensitivities of DISC**. The semi-relative sensitivities of DISC (*x*_21_) with respect to the rate constants of the formation of survival complex (*k*_9_), the formation of death complex (*k*_15_), the formation of DISC without TNFR1 (*k*_17 _), the caspase-8 activation (*k*_20_).

Following similar procedures mentioned above, we investigate the regulation of the apoptosis pathway. The DISC complex is essential for TNF-induced apoptosis and it is required for casepase-8 activation. To investigate the regulation of apoptosis, we identify the important reactions that regulate the formation of DISC by sensitivity analysis. The ranking of dynamic sensitivities of DISC based on the time-averaged semi-relative sensitivities is shown in Table 5. The key parameters -*k*_9_, *k*_15_, *k*_17_, and *k*_20 _- are identified and the dynamic sensitivities of DISC with respect to these four parameters are shown in Figure 10. The reaction of dissociation of DISC from the death receptor TNFR1 is essential for the following caspase-8 activation and its corresponding rate constant *k*_17 _is identify as an important parameter. This result is in agreement with the observation in an *in vitro *binding assay by Harper et al. [40].

**Table 5:** The ranking of semi-relative sensitivities of DISC

Rank	Parameter	Time-averaged semi-relative sensitivity	Percentage (%)
1	*k*_20_	0.1270	33.43
2	*k*_15_	0.0964	25.38
3	*k*_9_	0.0963	25.35
4	*k*_17_	0.0530	13.96

The dynamic sensitivities of DISC (*x*_21_) with respect to all parameters are ranked based on the time-averaged semi-relative sensitivities over the response time. The values of time-averaged semi-relative sensitivities and the corresponding ratios to the total semi-relative sensitivity are shown if the ratios are greater than 2%.

### Efficiency and accuracy

**Figure 11:**
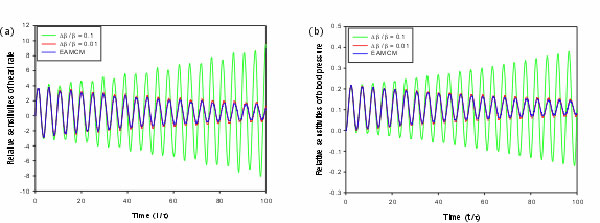
**The relative sensitivities obtained by the finite difference method and the EAMCM method**. a) The relative sensitivities of heart rate with respect to the uncontrolled average arterial blood pressure (*β*); b) The relative sensitivities of blood pressure with respect to *β*. The green and red lines are obtained by the finite difference method with spacing ratio 0.1 and 0.01, respectively. The blue line is obtained by the EAMCM method. The time is in dimensionless scale.

To verify the result obtained by the EAMCM algorithm, it is compared with the finite difference method using the dde23 as the DDE solver. The dde23 solver is available in MATLAB 6.5 and later. Forward difference is considered in the finite difference method. The dynamic sensitivities of these two systems mentioned above are solved by the finite difference method with spacing ratio 0.1 and 0.01, respectively. The relative parameter sensitivities of heart rate and blood pressure with respect to *β *obtained by the finite difference method with spacing ratio 0.1 and 0.01, respectively, and the EAMCM method are shown in Figure 11 as an illustration (another data is similar and not shown here). According to the definition of relative sensitivity, the theoretical value of relative sensitivity is obtained when the spacing ratio is approaching to zero. From Figure 11, the relative sensitivities obtained by the EAMCM are close to the theoretical values. We analyze the performance of the finite difference method and the EAMCM method for computing the dynamic sensitivities by measuring the number of evaluations of model equations. The results are shown in Table 6. The CPU time in second running by a 1.86 GHz Intel Xeon CPU with 4 GMB RAM is shown in the parenthesis for reference. Based on the comparison, the EAMCM program surely outperforms the finite difference method using dde23 solver. The EAMCM program can be accessed from http://www.che.ccu.edu.tw/~bioproc/index_english.files/page00064.htm and a brief manual can be found in the Additional file 2.

**Table 6:** The number of evaluations of model equations

System	Finite difference method with dde23 solver	EAMCM method	
Cardiovascular	441,112(160)	60,000(8)	
Apoptosis	311,464(246)	177,329(175)	

The number of evaluations of model equations for cardiovascular and apoptosis systems when computing all dynamic parameter and initial sensitivities by the finite difference method and the EAMCM method, respectively. The CPU time in second running by an 1.86 GHz Intel Xeon CPU with 4 GMB RAM is shown in the parenthesis.

## Conclusions

We extend the applicability of the adaptive direct-decoupled algorithm for ODE models to DDE models and include the implementation of automatic differentiation technique among it. The most attractive feature of the EAMCM program is minimal user intervention that can reduce the human effort required for solving the dynamic sensitivities of complex biological systems and reduce the number of human errors introduced. EAMCM requires the user to supply only the model equations at run-time to compute dynamic sensitivities of DDE models. The evaluation of sensitivity equations is done automatically by automatic differentiation technique along with the inevitable evaluation of model equations. The computations of partial derivatives and values of model equations simultaneously induce less overhead cost of computer time. The exact accuracy of the computed derivatives is achieved by the property of automatic differentiation. By compared with direct-coupled methods in theory, the adaptive direct-decoupled EAMCM algorithm is efficient, accurate, and easy to use for end users without programming background to do dynamic sensitivity analysis on complex biological systems with time-delays.

We illustrate the use of the EAMCM program in the sensitivity analysis of two DDE models: the cardiovascular control system and the TNF-*α *signal transduction network. The parameters for sympathetical and vagal control of heart rates are identified as key parameters in the cardiovascular control system. From the symmetry of dynamic effects of sympathetical and vagal control on heart rate obtained by sensitivity analysis, it reflects the sympathovagal balance in physiology. The TNF-*α *signal transduction network is a more complicated system than the first model and symbolic differentiation is unaffordable in this case to obtain the sensitivity equations. By using the EAMCM program, users can provide the model equations only for solving the dynamic sensitivities of the model. The formation of survival and death complexes are identified as the key reactions for the fragmentation of DNA via sensitivity analysis. This result reveals that the interplay between the components of the survival and apoptosis pathways plays an important role in the TNF-*α *signal transduction network.

## Methods

Delay differential equations (DDEs) arise in either natural or technological control problems for a large and important class of dynamical systems. This type of dynamical system now occupies a place of central importance in all areas of science and particularly in the biological sciences [45]. There are various kinds of delay differential equations. Here, we focus on equations with fixed, discrete delays, namely those of the form

(3) $$\frac{\text{d}x}{\text{d}t}=f(x(t),x(t-\tau_{1}),x(t-\tau_{2}),...,x(t-\tau_{r});\theta ),$$

where **x**(*t*) ∈ ℝ^*n *^is a vector of state variables, *θ *∈ ℝ^*p *^is a vector of parameters, *τ*_*i *_are positive time-delays, and *r *is the number of multiple delays. DDE models are similar to ODE models, but their evolution involves past values of the state variables. When giving initial conditions for ODE systems, we only need to specify the initial values of the state and input variables. In order to solve DDE systems, we have to look back to earlier values of **x **at every time step. Therefore, it is necessary to provide an initial function to specify the value of the solution before time *t *= 0. This function has to cover a period at least as long as the longest delay since we look back in time that far.

### DDE solver

Most DDEs do not have analytic solutions, so it is generally necessary to resort to numerical methods. We have presented an adaptive modified collocation method (AMCM) for computing the solution of autonomous ODE systems [21]. This method is easy to extend to compute the solution of simple scale DDE systems. To simplify discussion, we assume the DDE model consists of a set of DDEs with a single delay and expressed as

(4) $$\frac{\text{d}x}{\text{d}t}=f(x(t),x(t-\tau );\theta ),$$

where the delay *τ *is a positive constant. The initial function **x**(*t*) defined on the interval [-τ, 0] is set to **x**(0). Equation (4) can be reduced to ODEs by introducing a new input variable *y *for each delayed variablen *x*(*t *-*τ*) as

$$y(t)=\{\begin{array}{ll} x(0) & \text{if}\;t\leq \tau ,\\ x(t-\tau ) & \text{if}\;t>\tau , \end{array}$$

and can be solved by our ODE solver in the AMCM. The AMCM algorithm with piecewise linear polynomials as the shape functions solves the ODEs transformed from equation (4) for each subinterval [*t*_*j*-1_, *t*_*j*_], *t*_*i*-1 _≤ *t*_*j*-1 _<*t*_*j *_≤ *t*_*i *_by

(5) $$x(t_{j})=x(t_{j-1})+\frac{1}{2}\eta_{j}\{f(x(t_{j}),y(t_{j});\theta )+f(x(t_{j-1}),y(t_{j-1});\theta )\},$$

where *η*_*j *_is the step size in time *t*_*j *_. The adaptive ODE solver in the AMCM controls the step size automatically. The earlier values of **x **defined or computed on the interval [*t *- *τ*, *t*] have to be stored for **y **at every computation time *t*. Due to the automatic step size control, the solution at time *t *- *τ *may be not computed by the AMCM. To solve this problem, interpolation is used to generate the solution for the input variable *y *as follows:

(6) $$y(t)=x(t-\tau )=x(t_{a})+\frac{t-\tau -t_{a}}{t_{a+1}-t_{a}}[x(t_{a+1})-x(t_{a})],\;t_{a}<t-\tau <t_{a+1},$$

where *t*_*a *_is the time point that *x*(*t*_*a*_) has been computed, *t*_*a*+1 _is the next time point, and the step size *t*_*a*+1 _- *t*_*a *_is determined by the AMCM automatically.

### Dynamic sensitivity analysis

For a model described by equation (4), the *absolute parameter sensitivity s*(*x*_*i *_, *θ*_*j *_) of dependent variable *x*_*i *_∈ **x **with respect to a change in parameter *θ*_*j *_∈ *θ *is defined as

(7) $$s(x_{i},\theta_{j})=\underset{\Delta \theta_{j}\to 0}{\lim}\frac{x_{i}(t;\theta_{j}+\Delta \theta_{j})-x_{i}(t;\theta_{j})}{\Delta \theta_{j}}=\frac{\partial x_{i}(t;\theta )}{\partial \theta_{j}},$$

where *x*_*i*_(*t*; *θ*_*j *_+ Δ*θ*_*j*_) is the *i*^*th *^component of the solution of equation (4) with a change Δ*θ*_*j *_on the *j*^*th *^parameter and the others fixed. The absolute parameter sensitivity *s*(*x*_*i *_, *θ*_*j *_) is also defined as the first-order *local sensitivity *of *x*_*i *_with respect to *θ*_*j *_[46]. The term *local *refers to the fact that the value of *s*(*x*_*i *_, *θ*_*j *_) depends on the given set of values for the parameters *θ*. Under the assumption that the system responds linearly for small perturbations, *s*(*x*_*i *_, *θ*_*j *_) measures the ratio between the effect on *x*_*i *_and the variation of *θ*_*j *_. It is useful to consider the ratio between the relative effect on the output and the relative variation of a parameter when comparing different parameter sensitivities with respect to different parameters. *The relative parameter sensitivity S*(*x*_*i *_, *θ*_*j *_) of *x*_*i *_with respect to *θ*_*j *_, a dimensionless quantity, is defined as

(8) $$S(x_{i},\theta_{j})=\frac{\partial \ln x_{i}(t;\theta )}{\partial \ln\theta_{j}}=\frac{\theta_{j}}{x_{i}}s(x_{i},\theta_{j}).$$

Sometimes the use of relative parameter sensitivities has the difficulty of numerical instability caused by the division by zero when *x*_*i *_approaching zero. To address this problem, the *semi*-*relative parameter sensitivity *$\tilde{S}(x_{i},\theta_{j})$ of *x*_*i *_with respect to *θ*_*j *_is used and defined as

(9) $$\tilde{S}(x_{i},\theta_{j})=\theta_{j}s(x_{i},\theta_{j}).$$

Once the local sensitivity is known, the calculation of the relative sensitivity is straightforward. For brevity's sake, we limit our explanation on the absolute sensitivity only below. For an autonomous system describing by equation (4), the sensitivity equations are given as

(10) $$\frac{\text{d}s(x_{i}(t),\theta_{j})}{\text{d}t}=\sum_{k=1}^{n}\frac{\partial f_{i}}{\partial x_{k}(t)}s(x_{k}(t),\theta_{j})+\sum_{k=1}^{n}\frac{\partial f_{i}}{\partial x_{k}(t-\tau )}s(x_{k}(t-\tau ),\theta_{j})+\frac{\partial f_{i}}{\partial \theta_{j}},$$

where *f*_*i *_is the *i*^*th *^element of **f **. The AMCM algorithm is extended to compute the solution of equations (10), i.e., dynamic sensitivities of DDE systems. When the solution of a DDE system is obtained, the absolute dynamic sensitivity of *x*_*i *_with respect to *θ*_*j *_is computed by

(11) $$\begin{array}{c} s(x_{i}(t_{l}),\theta_{j})=s(x_{i}(t_{l-1}),\theta_{j})\\ +\frac{1}{2}\eta_{l}\left\{\sum_{k=1}^{n}\frac{\partial f_{i}(t_{l})}{\partial x_{k}(t_{l})}s(x_{k}(t_{l}),\theta_{j})+\sum_{k=1}^{n}\frac{\partial f_{i}(t_{l})}{\partial x_{k}(t_{l}-\tau )}s(x_{k}(t_{l}-\tau ),\theta_{j})+\frac{\partial f_{i}(t_{l})}{\partial \theta_{j}}\right\}\\ +\frac{1}{2}\eta_{l}\left\{\sum_{k=1}^{n}\frac{\partial f_{i}(t_{l-1})}{\partial x_{k}(t_{l-1})}s(x_{k}(t_{l-1}),\theta_{j})+\sum_{k=1}^{n}\frac{\partial f_{i}(t_{l-1})}{\partial x_{k}(t_{l-1}-\tau )}s(x_{k}(t_{l-1}-\tau ),\theta_{j})+\frac{\partial f_{i}(t_{l-1})}{\partial \theta_{j}}\right\}, \end{array}$$

where *η*_*l *_is the step size in time *t*_*l *_. Same as the AMCM algorithm, the extended algorithm (EAMCM) determines the step size automatically when computing the absolute dynamic sensitivity of *x*_*i *_.

The dynamic sensitivity in equation (8) reflects a relative relationship between the magnitudes of a parameter and a state variable at any time. We can define the integral value for the relative dynamic sensitivity over the whole time as a measure for ranking all sensitivities to identify the bottleneck reactions of the system. The time-averaged relative sensitivity is therefore defined as:

(12) $${\bar{S}}_{ij}=\frac{1}{t_{f}}\int_{0}^{t_{f}}|S(x_{i},\theta_{j})|\text{d}t,$$

where *t*_*f *_is the final time.

### Automatic differentiation

The main challenge in computing the solution of equation (10) is the evaluation of partial derivatives of **f **with respect to all state variables **x**, delay variables **x**(*t *- *τ*), and system parameters θ. One way to obtain the partial derivatives is to use symbolic differentiation tools, such as Maple and Mathematica, to perform the algebra of the differentiation. The explicit expression of the partial derivatives of **f **can be generated automatically. This is very useful because it saves the human effort and avoids the human errors in the analytical differentiation process. In principle, this approach gives exact values of the partial derivatives of **f **at the expense of high computation cost. Symbolic differentiation tools always generate lengthy formulas containing many common subexpressions that require considerable computation to evaluate. If only the values of the partial derivatives rather than the explicit expressions of the partial derivatives of **f **are needed, the simplest and common used approach is the numerical differentiation by finite difference approximation

(13) $$\frac{\partial f}{\partial x}=\underset{\Delta \text{x}\to 0}{\lim}\frac{f(x+\Delta x)-f(x)}{\Delta x}.$$

The main drawback of this approach is that the accuracy is hard to analyze. Another approach which can be used to evaluate partial derivatives is automatic differentiation. Automatic differentiation is a numerical computation of exact values of the partial derivatives without generating a formula for the partial derivatives and is much more effcient than symbolic differentiation. This approach is based on the fact that every function, no matter how complicated, can be represented by a well-formed expression that is a finite combination of elementary arithmetic operators, such as addition (+), subtraction (-), multiplication (*), division (/), and power (^), primitives, such as a constant or a state variable, and intrinsic functions, such as sin, cos, etc. Each elementary arithmetic operation involves at most two operands which either have been computed in a previous step or are primitives. The chain rule can be applied to each of elementary arithmetic operators as follows:

(14) $$\frac{\partial h(o_{1},o_{2})}{\partial x}=\frac{\partial h}{\partial o_{1}}\frac{\partial o_{1}}{\partial x}+\frac{\partial h}{\partial o_{2}}\frac{\partial o_{2}}{\partial x},$$

where *h *indicates the function for each elementary arithmetic operator and *o*_*i *_is the *i*^*th *^operand. The derivative of each primitive and each intrinsic function would have to know. By applying the chain rule recursively to each elementary arithmetic operator and each intrinsic function, the partial derivatives of a function can be computed exactly and in a completely mechanical fashion.

## EAMCM algorithm

The proposed algorithm EAMCM is shown as follows:

Algorithm EAMCM

**Input**:

1. A set of *n *delay differential equations $\dot{x}$ = **f **(**x, y**) with *n *dependent variables *x*_*i *_, *i *= 1, ..., *n *and *m *time-delay variables *y*_*i *_≡ *x*_*j *_(*t *- *τ*), *i *= 1, ..., *m*, *j *∈ [1, *n*].

2. Two order sets **x**_0 _= {*x*_*i *_(0)|*i *= 1, ..., *n*} and **Φ**_0 _= {*ϕ*_*ij *_(0)|*i *= 1, ..., *n*, *j *= 1, ..., *m*}.

3. An order set *T *= {*t*_1_, ..., *t*_*k*_} of sampling points, *t*_*i*_, 1 ≤ *i *≤ *k *is the sampling time of the solution of each DDE, *k *is the number of sampling points.

4. A tolerance *ε*.

**Output**: The dynamic sensitivities of dependent variables at each sampling time.

• For each sampling time *t*_*i *_in *T*.

1. *η*_*j *_← *t*_*i *_- *t*_*i*-1_, *d*_*t *_← 0, **x**^*c *^← **x**(*t*_*i*-1 _), **Φ**^*c *^← **Φ**(*t*_*i*-1_).

2. Repeat the following steps until *d*_*t *_= *t*_*i *_- *t*_*i*-1_.

(a) **x**^*p *^← **x**^c ^, **y**^p ^← **x**(t_*i*-1 _+ *d*_*i *_- *τ*), **Φ**^*p *^← **Φ**^*c *^.

(b) If *t*_*i*-1 _>*τ *, then **Φ**^*d *^← **Φ**(*t*_*i*-1_+ *d*_*i *_- *τ *); otherwise, **Φ**^*d *^← **Φ**_0_.

(c) Evaluate the Jacobin matrix by automatic differentiation $A\leftarrow \left[\frac{\partial f\text{(}x^{p}\text{,}y^{p}\text{)}}{\partial x},\frac{\partial f\text{(}x^{p}\text{,}y^{p}\text{)}}{\partial y}\right]$.

(d) Compute the upper bound μ of the value of ||**A**||_2 _by $\sqrt{n(m+n)}∥A{∥}_{\Delta },∥A{∥}_{\Delta }\equiv \underset{i,j}{\max}\left|a_{ij}\right|$.

(e) If μ * *ε *≥ 1, it means the DDEs are stiff, then exit this algorithm.

(f) If μ * *η*_*j *_> 1, then *η*_*j *_← 0.9/*μ*.

(g) Call the Iteration algorithm to compute the value of **x**^*c *^stepped forward *η*_*j *_from **x**^*p *^.

(h) Call the IterationOfSensitivity algorithm to compute the value of the sensitivity matrix **Φ**^*c *^stepped forward *η*_*j *_from **Φ**^*p*^.

(i) If the Iteration and IterationOfSensitivity algorithms succeed in computing **x**^*c *^and **Φ**^*c *^respectively, then *d*_*t *_← *d*_*t *_+ *η*_*j *_and *η*_*j *_← *t*_*i *_- *t*_*i*-1_*-d*_*t *_; otherwise exist this algorithm.

3. Save the value of the sensitivity matrix **Φ**^*c *^as **Φ**(*t*_*i*_).

• return Φ(*t*_*i*_), *i *= 1, ..., *k*.

End of Algorithm EAMCM

**Algorithm **Iteration

Input:

1. A set of *n *delay differential equations $\dot{x}$ = **f **(**x, y**), **y**(*t*) ≡ **x**(*t *- *τ *).

2. **x**(*t*), **y**(*t*), *η*_*j *_, and the iteration limitation.

**Output**: **x**(*t *+ *η*_*j *_).

1. Evaluate the value of **f **(**x**(*t*), **y**(*t*)).

2. **x**(*t *+ *η*_*j*_) ← **x**(*t*) + **f **(**x**(*t*), **y**(*t*)) * *η_j_*.

3. **y**(*t *+ *η_j_*) ← **x**(*t *+ *η_j _*- τ).

4. Repeat the following steps until the iteration limitation is reached or the value of **x**(*t*+*η_t_*) is convergent.

(a) Evaluate the value of **f **(**x**(*t *+ *η_j_*), **y**(*t *+ *η_j _*)).

(b) **x**(*t *+ *η_j_*) ← **x**(*t*) + 0.5 * η_j _* (**f **(**x**(*t*), **y**(*t*)) + **f** (**x**(*t *+ *η_j_*), **y**(*t *+ *η_j_*))).

5. If the iteration limitation is reached, then exit this algorithm; otherwise, return **x**(*t *+ *η_j_*).

**End of Algorithm **Iteration

**Algorithm **IterationOfSensitivity

**Input**:

1. A set of *n *delay differential equations $\dot{x}$ = **f **(**x, y**), **y**(*t*) ≡ **x**(*t *- *τ*).

2. *η_j _*and the iteration limitation.

3. Two vectors of dependent variables **x**(*t*) at time *t *and **x**(*t *+ *η_j_*) at time *t *+ *η_j _*.

4. Two sensitivity matrices **Φ**(**x**(*t*)) and **Φ**(**y**(*t*)).

**Output**: The sensitivity matrix **Φ**(**x**(*t *+ *η*_*j *_)).

1. Evaluate $A=\frac{\partial f(x(t),y(t))}{\partial x}$, $B\;=\;\frac{\partial f(x(t),y(t))}{\partial y}$, and $C=\frac{\partial f(x(t),y(t))}{\partial \theta }$ by automatic differentiation.

2. Evaluate the derivative $\dot{\Phi }$(**x**(*t*)) = **AΦ**(**x**(*t*)) + **BΦ**(**y**(*t*)) + **C**.

3. **Φ**(**x**(*t *+ *η*_*j*_)) = **Φ**(**x**(*t*)) + $\dot{\Phi }$(**x**(*t*)) * *η_j _*.

4. Evaluate $A=\frac{\partial f(x(t+\eta j),y(t+\eta j))}{\partial x}$, $B\;=\;\frac{\partial f(x(t+\eta j),y(t+\eta j))}{\partial y}$, and $C\;=\;\frac{\partial f(x(t+\eta j),y(t+\eta j))}{\partial \theta }$ by automatic differentiation.

5. Repeat the following steps until the iteration limitation is reached or the value of Φ(**x**(t + *η*_*j*_)) is convergent.

(a) Evaluate the derivative $\dot{\Phi }$(**x**(*t *+ *η*_*j*_)) = **AΦ**(**x**(*t *+ *η*_*j*_)) + **BΦ**(**y**(*t *+ *η*_*j*_)) + **C**.

(b) Evaluate the new value of **Φ**(**x**(*t *+ *η*_*j*_)) by

$$\Phi (x(t+\eta_{j}))=\Phi (x(t))\;+\;0.\text{5}*\eta_{j}*(\dot{\Phi }(x(t+\eta_{j}))+\dot{\Phi }(x(t))).$$

6. If the iteration limitation is reached, then exit this algorithm; otherwise, return **Φ**(**x**(*t *+ *η_j_*)).

**End of Algorithm **IterationOfSensitivity

## Competing interests

The authors declare that they have no competing interests.

## Authors' contributions

WHW developed and implemented the algorithm and drafted the manuscript. FSW conceived of the study, participated in its design and coordination, and helped to draft the manuscript. MSC assisted in developing the algorithm and finalizing the manuscript. All authors read and approved the final manuscript.

## Supplementary Material

Additional file 1 **TNF-*α *signal transduction model**. This file includes the set of delay differential equations of the TNF-*α *signal transduction model, all of the relevant definitions of state variables, and the nominal values of parameters appearing in the delay differential equations. Click here for file

Additional file 2 **Manual of EAMCM program**. This document provides a brief introduction to the extended adaptive modified collocation method (EAMCM) that we implemented to compute the solution and dynamic sensitivities of ordinal differential equation (ODE) and delay differential equation (DDE) systems. Click here for file
