# Supplementary material for: Sensitivity analysis of dynamic biological systems with time-delays
Source: BMC Bioinformatics. 2010 Oct 15;11(Suppl 7):S12. doi: 10.1186/1471-2105-11-S7-S12 (PMC2957680; doi:10.1186/1471-2105-11-S7-S12)
Supplement: Additional file 1 — TNF-α signal transduction model. This file includes the set of delay differential equations of the TNF-α signal transduction model, all of the relevant definitions of state variables, and the nominal values of parameters appearing in the delay differential equations. [file 1471-2105-11-S7-S12-S1.pdf]

Supplementary information
Sensitivity analysis of dynamic biological systems with time-delays

Wu Hsiung Wu[1], Feng Sheng Wang*[2] and Maw Shang Chang[1]

[1]Department of Computer Science and Information Engineering, National Chung Cheng University, Chiayi 62102, Taiwan
[2]Department of Chemical Engineering, National Chung Cheng University, Chiayi 62102, Taiwan

Email: Wu Hsiung Wu - wwh@cs.ccu.edu.tw; Feng Sheng Wang*- chmfsw@ccu.edu.tw; Maw Shang Chang - mschang@cs.ccu.edu.tw;

*Corresponding author

The TNF-α signal transduction model analyzed in this paper was developed by Rangamani et al. [1]. This model consists of 31 delay differential equations which include 29 parameters. The DDEs are derived according to the law of mass balance and reaction kinetics and the parameters are the kinetic rate constants. The state variables are the concentration of the molecules in the survival and apoptosis pathways and the input variable is the concentration of TNF-α that stimulates the signal transduction pathways. The output variable is the concentration of fragmented DNA, which can be as a marker for apoptosis. The fragmented DNA is defined as the fraction of DNA sites that have been attacked by the activity of effector caspase.

Model equations

$$
\begin{aligned}
\frac{\mathrm{d}x_1}{\mathrm{d}t} &= -k_1x_1x_2 + k_2x_3 \\
\frac{\mathrm{d}x_2}{\mathrm{d}t} &= -k_1x_1x_2 + k_2x_3 + k_{17}x_{18} + k_{11}x_{11} \\
\frac{\mathrm{d}x_3}{\mathrm{d}t} &= k_1x_1x_2 - k_2x_3 - k_3x_3x_4 + k_4x_5 \\
\frac{\mathrm{d}x_4}{\mathrm{d}t} &= -k_3x_3x_4 + k_4x_5 + k_{11}x_{11} + k_{20}x_{21} \\
\frac{\mathrm{d}x_5}{\mathrm{d}t} &= k_3x_3x_4 - k_4x_5 - k_5x_5x_6 + k_6x_7 \\
\frac{\mathrm{d}x_6}{\mathrm{d}t} &= -k_5x_5x_6 + k_6x_7 + k_{11}x_{11} + k_{20}x_{21} \\
\frac{\mathrm{d}x_7}{\mathrm{d}t} &= k_5x_5x_6 - k_6x_7 - k_7x_7x_8 + k_8x_9
\end{aligned}
$$

$$\frac{\mathrm{d}x_8}{\mathrm{d}t} = -k_7 x_7 x_8 + k_8 x_9 + k_{11} x_{11} + k_{20} x_{21}$$
$$\frac{\mathrm{d}x_9}{\mathrm{d}t} = k_7 x_7 x_8 - k_8 x_9 - k_9 x_9 x_{10} + k_{10} x_{11} - k_{15} x_9 x_{17} + k_{16} x_{18}$$
$$\frac{\mathrm{d}x_{10}}{\mathrm{d}t} = -k_9 x_9 x_{10} + k_{10} x_{11} + k_{14} x_{14}$$
$$\frac{\mathrm{d}x_{11}}{\mathrm{d}t} = k_9 x_9 x_{10} - k_{10} x_{11} + k_{11} x_{11}$$
$$\frac{\mathrm{d}x_{12}}{\mathrm{d}t} = -k_{12} x_{12} x_{13} + k_{13} x_{14} + k_{11} x_{11}$$
$$\frac{\mathrm{d}x_{13}}{\mathrm{d}t} = -k_{12} x_{12} x_{13} + k_{13} x_{14} + k_{28} x_{16} x_{31}$$
$$\frac{\mathrm{d}x_{14}}{\mathrm{d}t} = k_{12} x_{12} x_{13} - k_{13} x_{14} - k_{14} x_{14}$$
$$\frac{\mathrm{d}x_{15}}{\mathrm{d}t} = k_{14} x_{14}$$
$$\frac{\mathrm{d}x_{16}}{\mathrm{d}t} = k_{14} x_{14} - k_{28} x_{16} x_{31}$$
$$\frac{\mathrm{d}x_{17}}{\mathrm{d}t} = -k_{15} x_9 x_{17} + k_{16} x_{18} + k_{20} x_{21}$$
$$\frac{\mathrm{d}x_{18}}{\mathrm{d}t} = k_{15} x_9 x_{17} - k_{16} x_{18} - k_{17} x_{18}$$
$$\frac{\mathrm{d}x_{19}}{\mathrm{d}t} = k_{17} x_{18} - k_{18} x_{19} x_{20} + k_{19} x_{21}$$
$$\frac{\mathrm{d}x_{20}}{\mathrm{d}t} = -k_{18} x_{19} x_{20} + k_{19} x_{21}$$
$$\frac{\mathrm{d}x_{21}}{\mathrm{d}t} = k_{18} x_{19} x_{20} - k_{19} x_{21} - k_{20} x_{21}$$
$$\frac{\mathrm{d}x_{22}}{\mathrm{d}t} = k_{20} x_{21} - k_{21} x_{22} x_{23} + k_{22} x_{24} + k_{23} x_{24}$$
$$\frac{\mathrm{d}x_{23}}{\mathrm{d}t} = -k_{21} x_{22} x_{23} + k_{22} x_{24}$$
$$\frac{\mathrm{d}x_{24}}{\mathrm{d}t} = k_{21} x_{22} x_{23} - k_{22} x_{24} - k_{23} x_{24}$$
$$\frac{\mathrm{d}x_{25}}{\mathrm{d}t} = k_{23} x_{24} - k_{27} x_{25} x_{27} - k_{24} x_{25} x_{29} + k_{25} x_{30} + k_{26} x_{30}$$
$$\frac{\mathrm{d}x_{26}}{\mathrm{d}t} = k_{26} x_{30}$$
$$\frac{\mathrm{d}x_{27}}{\mathrm{d}t} = k_{29} x_{16}(t-\tau) - k_{27} x_{25} x_{27}$$
$$\frac{\mathrm{d}x_{28}}{\mathrm{d}t} = k_{27} x_{25} x_{27}$$
$$\frac{\mathrm{d}x_{29}}{\mathrm{d}t} = -k_{24} x_{25} x_{29} + k_{25} x_{30}$$
$$\frac{\mathrm{d}x_{30}}{\mathrm{d}t} = k_{24} x_{25} x_{29} - k_{25} x_{30} - k_{26} x_{30}$$
$$\frac{\mathrm{d}x_{31}}{\mathrm{d}t} = k_{29} x_{16}(t-\tau) - k_{28} x_{16} x_{31}$$

State variables and initial values

Variable	species	Initial value (nM)
x_1	TNF-α	1
x_2	TNFR1	100
x_3	TNF-α/TNFR1	0
x_4	TRADD	150
x_5	TNF-α/TNFR1/TRADD	0
x_6	TRAF2	100
x_7	TNF-α/TNFR1/TRADD/TRAF2	0
x_8	RIP-1	100
x_9	TNF-α/TNFR1/TRADD/TRAF2/RIP1	0
x_{10}	IKK	100
x_{11}	TNF-α/TNFR1/TRADD/TRAF2/RIP1/IKK (survival complex)	0
x_{12}	IKK*	0
x_{13}	IκB/NF-κB	250
x_{14}	IκB/NF-κB/IKK*	0
x_{15}	IκB-P	0
x_{16}	NF-κB	0
x_{17}	FADD	100
x_{18}	TNF-α/TNFR1/TRADD/TRAF2/RIP1/FADD (death complex)	0
x_{19}	TRADD/TRAF2/RIP1/FADD	0
x_{20}	Caspase-8	80
x_{21}	TRADD/TRAF2/RIP1/FADD/Caspase-8 (DISC)	0
x_{22}	Caspase-8*	0
x_{23}	Caspase-3	200
x_{24}	Caspase-8*/Caspase-3	0
x_{25}	Caspase-3*	0
x_{26}	DNA fragmentation	0
x_{27}	cIAP	0
x_{28}	Caspase-3*/cIAP	0
x_{29}	DNA (intact)	800
x_{30}	Caspase-3*/DNA	0
x_{31}	IκB	0

Table S1: **State variables and their initial values.** The first column is the state variable name, the second column is the corresponding species, and the third column is the initial value in nM for each state variable. The initial values are obtained from Cho et al. [2].

Kinetic parameters

Kinetic Parameter	Value	Kinetic Parameter	Value
k_1	1.11×10^{-2}	k_{16}	7.5×10^{-5}
k_2	7.5×10^{-5}	k_{17}	2.22×10^{-2}
k_3	1.11×10^{-2}	k_{18}	3×10^{-2}
k_4	7.5×10^{-5}	k_{19}	1.2×10^{-2}
k_5	1.11×10^{-2}	k_{20}	6×10^{-3}
k_6	7.5×10^{-5}	k_{21}	6×10^{-3}
k_7	1.11×10^{-2}	k_{22}	3.6×10^{-3}
k_8	7.5×10^{-5}	k_{23}	6
k_9	1.11×10^{-2}	k_{24}	1.11×10^{-2}
k_{10}	7.5×10^{-5}	k_{25}	7.5×10^{-5}
k_{11}	2.22×10^{-2}	k_{26}	2.22×10^{-2}
k_{12}	8.4×10^{-4}	k_{27}	3×10^{-2}
k_{13}	7.5×10^{-5}	k_{28}	45
k_{14}	2.22×10^{-2}	k_{29}	1.05×10^{-1}
k_{15}	1.11×10^{-2}		

Table S2: **Kinetic parameters.** The nominal values of kinetic parameters. Units for kinetic parameters are M and mim.

References

1. Rangamani P, Sirovich L: **Survival and apoptotic pathways initiated by TNF-α: modeling and predictions.** *Biotechnology and Bioengineering* 2007, **97**:1216–1229.
2. Cho KH, Shin SY, Kolch W, Wolkenhauer O: **Experimental design in systems biology, based on parameter sensitivity analysis using a Monte Carlo method: a case study for the TNFα-mediated NF-κB signal transduction pathway**. *Simulation* 2003, **79**:726–739.
