# Supplementary material for: Sensitivity analysis of dynamic biological systems with time-delays
Source: BMC Bioinformatics. 2010 Oct 15;11(Suppl 7):S12. doi: 10.1186/1471-2105-11-S7-S12 (PMC2957680; doi:10.1186/1471-2105-11-S7-S12)
Supplement: Additional file 2 — Manual of EAMCM program. This document provides a brief introduction to the extended adaptive modified collocation method (EAMCM) that we implemented to compute the solution and dynamic sensitivities of ordinal differential equation (ODE) and delay differential equation (DDE) systems. [file 1471-2105-11-S7-S12-S2.pdf]

Supplementary information
Sensitivity analysis of dynamic biological systems with time-delays

Wu Hsiung Wu[1], Feng Sheng Wang[*2] and Maw Shang Chang[1]

[1]Department of Computer Science and Information Engineering, National Chung Cheng University, Chiayi 62102, Taiwan

[2]Department of Chemical Engineering, National Chung Cheng University, Chiayi 62102, Taiwan

Email: Wu Hsiung Wu - wwh@cs.ccu.edu.tw; Feng Sheng Wang* - chmfsw@ccu.edu.tw; Maw Shang Chang - mschang@cs.ccu.edu.tw;

* Corresponding author

Introduction

This document provides a brief introduction to the extended adaptive modified collocation method (EAMCM) that we implemented to compute the solution and dynamic sensitivities of ordinal differential equation (ODE) and delay differential equation (DDE) systems. The EAMCM program is implemented with mixed-language programming employing Microsoft Visual BASIC, Visual C++, and Intel FORTRAN. Mixed-language programming is very useful for many applications which can take advantage of the salient features of different programming languages. The graphic user interface (GUI) of the program is implemented by Microsoft Visual BASIC. It contains a wide variety of objects for use in the GUI and is easy to use for improving the user interface design. The input of differential equations and Jacobian matrix or gradient is not an easy job. They are provided through mathematical expressions and parsed by the compiler embedded in the EAMCM program. The parser to checking the syntax errors is implemented by Visual C++, because it provides object-oriented capabilities which should help us in developing high quality software. In addition to the parser, we use C++ object-oriented programming to design the new method for computing dynamic sensitivities of DDEs. We have developed many efficient and specifiable high-precision source codes implemented by Intel FORTRAN for computing the solution of optimization problems. FORTRAN programming language is efficient in the computation of mathematical arrays and is easy to ensure that the precision of floating-point numbers. To save our development time, these FORTRAN codes are compiled to a run-time library and linked into our program.

Installation

The EAMCM program can be accessed from http://www.che.ccu.edu.tw/~bioproc/index_english.files/page00064.htm. This program should be run on Microsoft Windows XP SP2 (X86) or later Service Packs. To install EAMCM program, uninstall the zip archive in a directory of your choice and execute the setup.exe directly. When the program is installing, the installer will check the running environment of your computer and connect to the web site of Microsoft to update your operating system automatically if it is required for the program. The installer will create a new shortcut of the program on the desktop in Windows when it finishes the installation. Two examples, cardiovascular system and apoptosis system, that we show in the paper are given for demo. The input text files for these two examples are included in the program package and built in the same directory of your choice for the execution file when the program is installed.

Starting EAMCM program

To use EAMCM , double click the shortcut of the program on the desktop. After invoking the EAMCM program, the main window will start as follows:

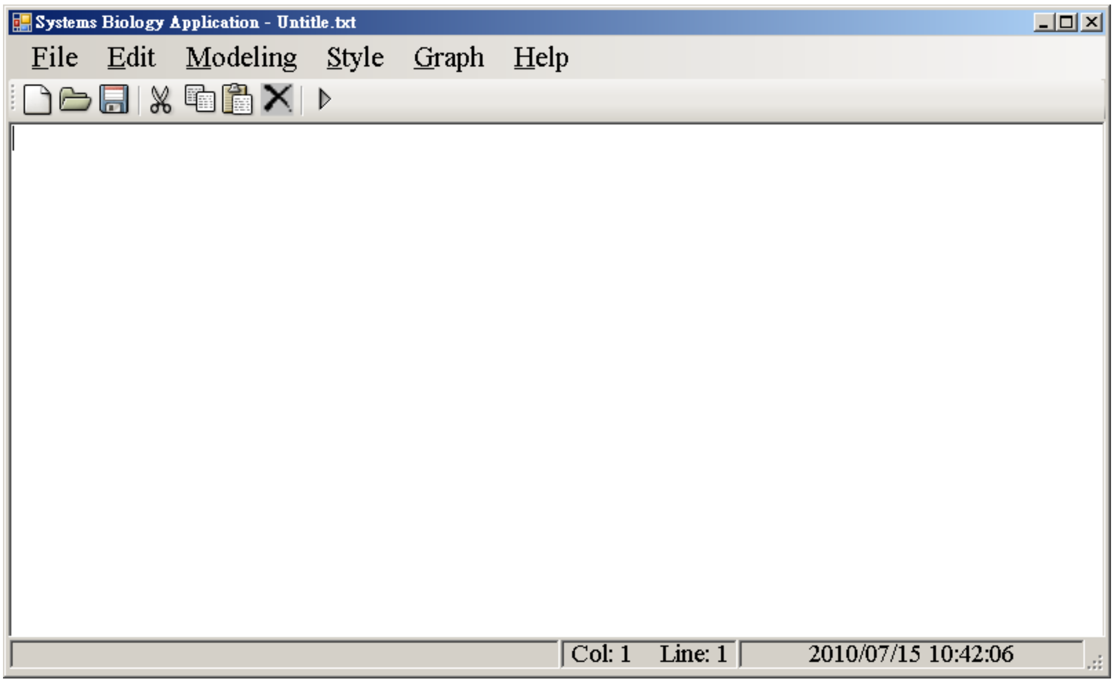

The main window offers access to the main features of EAMCM program. The File menu is used to manipulate the input text file, such as open an existing file, save the input text file, print the input text file, etc. The Modeling menu provides different forms to help user to create a new model for ODE or DDE systems and analyze different models. The Graph menu offers access to the plots of output data.

Loading a model File

Use File->Open command to select an existing model file, CardiovascularSystem_fr10_AutoDiff, from the file selection dialog. The content of the text file will appear in the display area of the main window as follows:

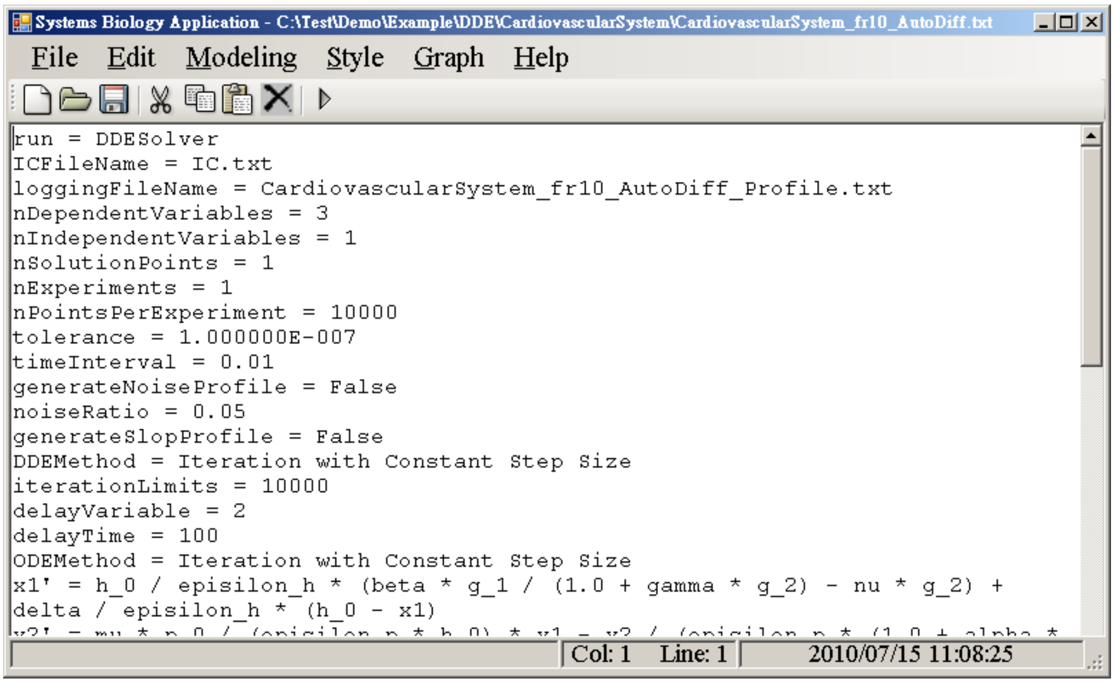

You can modify the content of the file directly if some of parameters are changed, but it is not suggested for a new user. When a model text file is generated, the corresponding solver kept in the text file can be invoked by just pressing the "run" button ▷ in the tool strip. When you close the EAMCM program or load another models, you will be requested to save the current model.

Create a new model

To compute the solution and dynamic sensitivities of an ODE or DDE model, model equations (ODEs or DDEs) and some information about the model should be provided through an input text file. The contents of the input text file can be generated automatically and modified by the assistance of an input form. Use Modeling->Solver->DDESolver to create a new DDE model. An input form will pop up for a DDE model as follows:

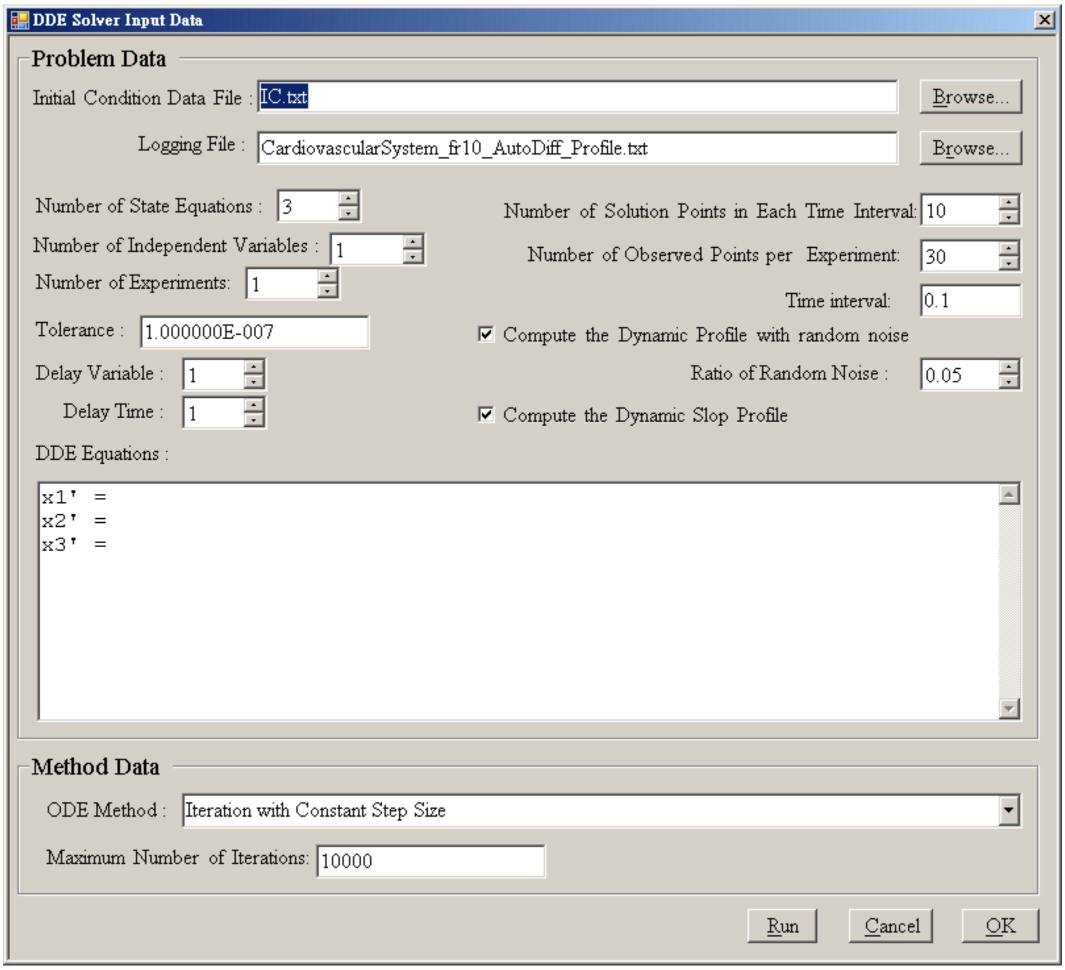

Default values for the parameters are used if a new model is created. If an existing model is opened already, its content will be showed in the input form. The values of the parameters of the model can be input by the editing box. Three template differential equations are shown in the "DDE Equations" text box and the number of equations can be extended by appending differential equations in the text box. The words beginning with the letter x and p, followed by a number, are reserved for the name of state variables and parameters, respectively. The initial values of state variables and parameters should be input such as x1 = 1.25 and p1 = 2.51, etc. x1' in the left hand side of equal sign indicates the first differential equations. Users can define their self-defined variables, except the predefined variables, in the differential equations. Variable names follow the same rules of programming language. The

variable name is case-sensitive. A valid variable name starts with a letter or underscore, followed by any number of letters, numbers, or underscores. The words beginning with the letter J, followed by a left parenthesis, are reserved for the name of the elements of Jacobian matrix. When the input form is filled and the OK button is pressed, a template file will be generated automatically and show in the display area of the main window. A snap shot of a default template file is shown in the following:

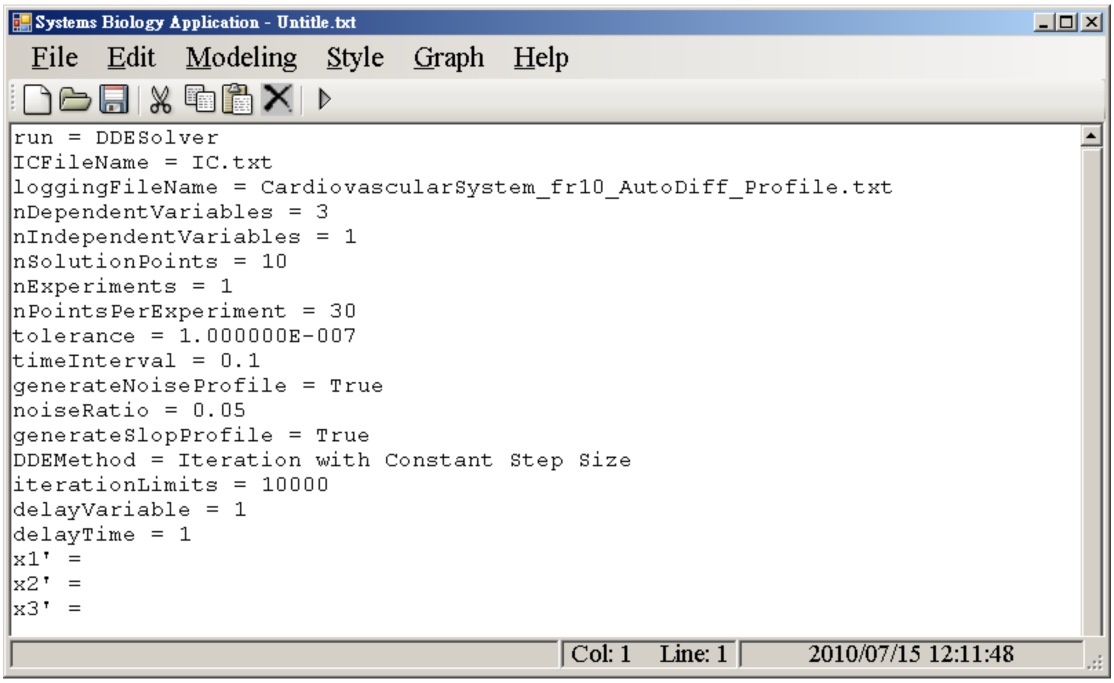


```
run = DDESolver
ICFileName = IC.txt
loggingFileName = CardiovascularSystem_fr10_AutoDiff_Profile.txt
nDependentVariables = 3
nIndependentVariables = 1
nSolutionPoints = 10
nExperiments = 1
nPointsPerExperiment = 30
tolerance = 1.000000E-007
timeInterval = 0.1
generateNoiseProfile = True
noiseRatio = 0.05
generateSlopProfile = True
DDEMethod = Iteration with Constant Step Size
iterationLimits = 10000
delayVariable = 1
delayTime = 1
x1' =
x2' =
x3' =
```

If the form is filled fully, you can run the DDE solver directly by pressing the "Run" button in the input form. The contents of the template text file can also be modified directly in the display area of the main window. The result data will be shown in a pop window and the plots for the data are generated on the background and can be viewed using Graph menu later.

Plotting the data

EAMCM program provide many ways to customize your plots. When the output data is generated, the plots for the data are generated automatically on the background and saved using the logging file name that user input with a "png" extension name. You can select them using the Graph->Open command from the file selection dialog box. If many state variables exist in the model, the Graph->Create command allows you to choose which data to plot. For example, you can choose to plot the time profile for all state variables, only some interesting state variables, or all state variables excluding some state variables in the apoptosis model. In the following dialog, the time profile for the x16 and x31 variables is choose to plot and the result is shown when the "Create" button in the dialog box is pressed.

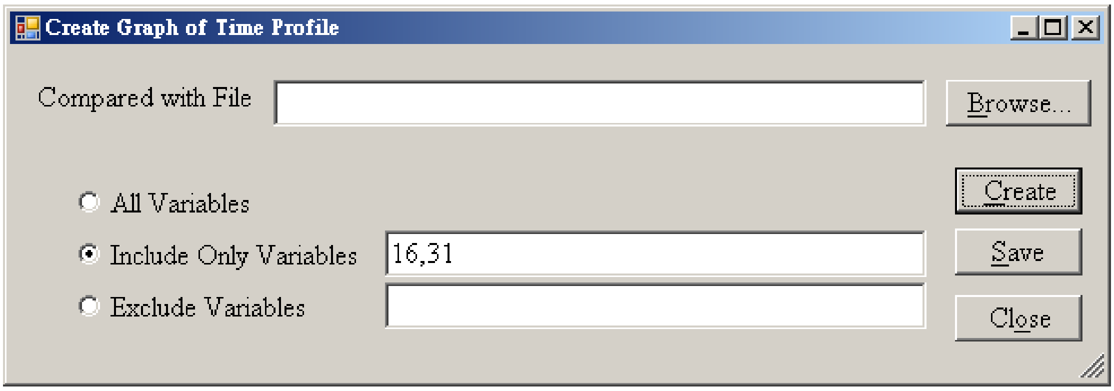

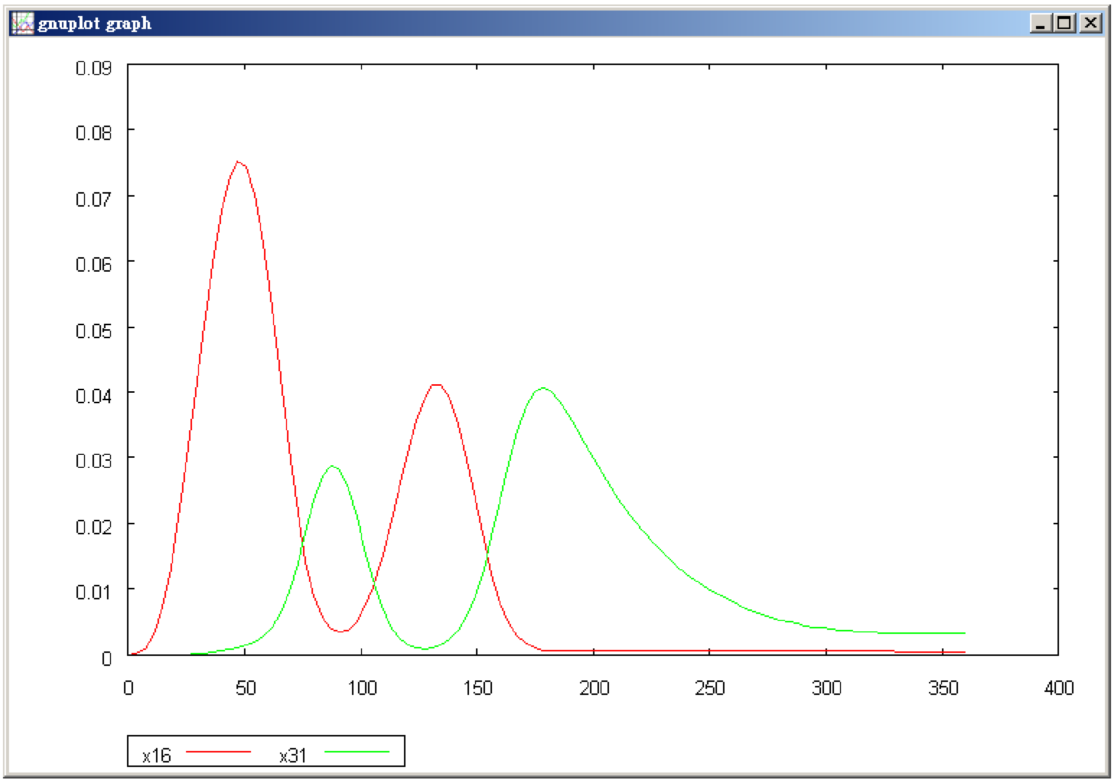

Example - Dynamic sensitivity analysis of apoptosis model

Apoptosis system is a complex DDE model and takes a few minutes to compute the solution and plot the time profile. The output data is stored in a file named as the user specified and shown directly in a popup window when it is finished. The graph for the time profile is generated on the background and can be shown later by the Open command in the Graph menu. The input text files for the apoptosis model are included in the program package and built in the same directory for the execution file when the program is installed.

Use the File->Open command to open "Apoptosis_Sensitivity_AutoDiff.txt" file and press the "run" button in the tool strip. The output data is shown in a pop-up window as follows:

Sensitivity Data Viewer - Apoptosis_Sensitivity_AutoDiff_Profile.txt

T...	S(x1,p1)	S(x1,p2)	S(x1,p3)	S(x1,p4)	S(x1,p5)
0....	0.000000000000000	0.000000000000000	0.000000000000000	0.000000000000000	0.00000000000000[illegible]
1....	-0.364965534536747	0.000011482718835	-0.000005826993206	0.000000000115082	-0.00000002959874
2....	-0.242165773161441	0.000012968709691	-0.000012326080014	0.000000000503298	-0.00000048053760
3....	-0.120873118948452	0.000008383080893	-0.000011424356473	0.000000000711369	-0.00000110517394
4....	-0.053678711360044	0.000004342053024	-0.000007556991601	0.000000000633880	-0.00000118149608
5....	-0.022353222675754	0.000002000253365	-0.000004137729378	0.000000000438309	-0.00000085151007
6....	-0.008935680519503	0.000000857979125	-0.000002008509721	0.000000000258422	-0.00000048753029
7....	-0.003472275441135	0.000000350986426	-0.000000898726553	0.000000000136785	-0.00000024163347[illegible]
8....	-0.001321484645431	0.000000138867809	-0.000000379832489	0.000000000067060	-0.00000010892925[illegible]
9....	-0.000494972071953	0.000000053607359	-0.000000154050913	0.000000000031069	-0.00000004603624
10...	-0.000183068088171	0.000000020308924	-0.000000060600093	0.000000000013787	-0.00000001859376
11...	-0.000067017652155	0.000000007581212	-0.000000023291653	0.000000000005915	-0.00000000726772
12...	-0.000024326107881	0.000000002796612	-0.000000008791869	0.000000000002470	-0.00000000277230[illegible]
13...	-0.000008766814834	0.000000001021622	-0.000000003271315	0.000000000001009	-0.00000000103798[illegible]
14...	-0.000003140127937	0.000000000370178	-0.000000001203106	0.000000000000405	-0.00000000038300
15...	-0.000001118785199	0.000000000133208	-0.000000000438238	0.000000000000160	-0.00000000013968
16...	-0.000000396760608	0.000000000047651	-0.000000000158351	0.000000000000062	-0.00000000005045
17...	-0.000000140129388	0.000000000016958	-0.000000000056827	0.000000000000024	-0.00000000001808
18...	-0.000000049311117	0.000000000006008	-0.000000000020274	0.000000000000009	-0.00000000000643
19...	-0.000000017295770	0.000000000002120	-0.000000000007196	0.000000000000003	-0.00000000000227
20...	-0.000000006048586	0.000000000000745	-0.000000000002543	0.000000000000001	-0.00000000000080
21...	-0.000000002109628	0.000000000000261	-0.000000000000895	0.000000000000000	-0.00000000000028
22...	-0.000000000734005	0.000000000000091	-0.000000000000314	0.000000000000000	-0.00000000000009
[illegible]	[illegible]	[illegible]	[illegible]	[illegible]	[illegible]

The execution time is shown in the left bottom of the main window.

We will show how to create the plot of parameter sensitivities of x16 with respect to parameter 9, 11, 12, 14, 15, 28, and 29. Use the Graph->Create command to invoke the "Create" dialog box and select the parameter sensitivities as follows:

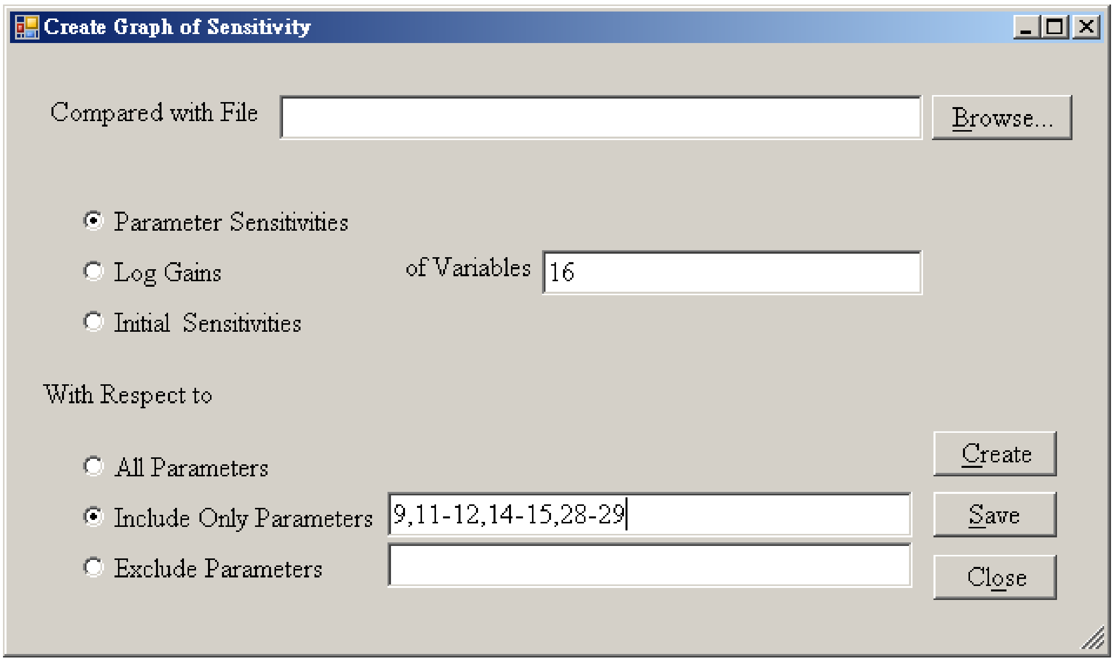

Input the number of state variables and parameters, then press the "Create" button in the dialog box. The output is shown as follows:

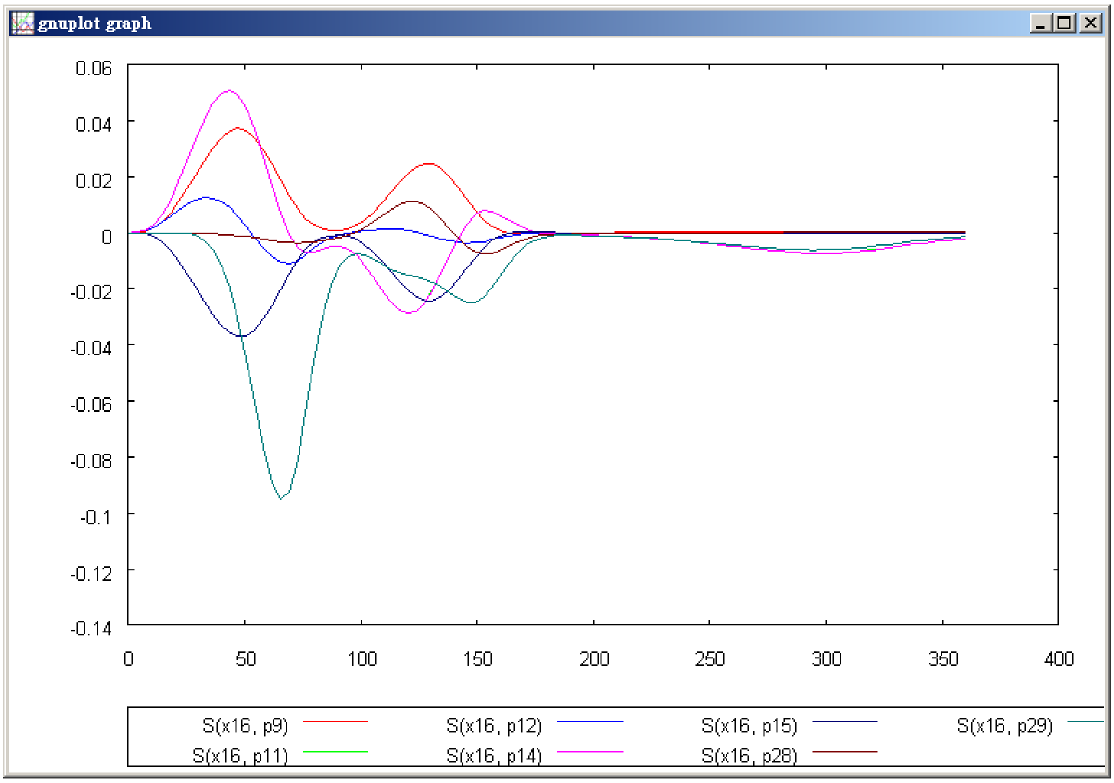

If you want to save the output graph, press the "Save" button in the dialog box. The "Create" dialog box also provides the function of comparison for two different data

files. For example, the sensitivities of x16 with respect to k9 in the apoptosis model for different values of k9 are interested to you. The parameter sensitivities of all state variables of the apoptosis DDE model for k9 = 0.111 are computed and saved in the "Apoptosis_Sensitivity_AutoDiff_Profile_9.txt" file. The current model for k9 = 0.0111 is opened and shown in the display area of main window. Use the Graph->Create command to invoke the "Create" dialog box and input the data as follows:

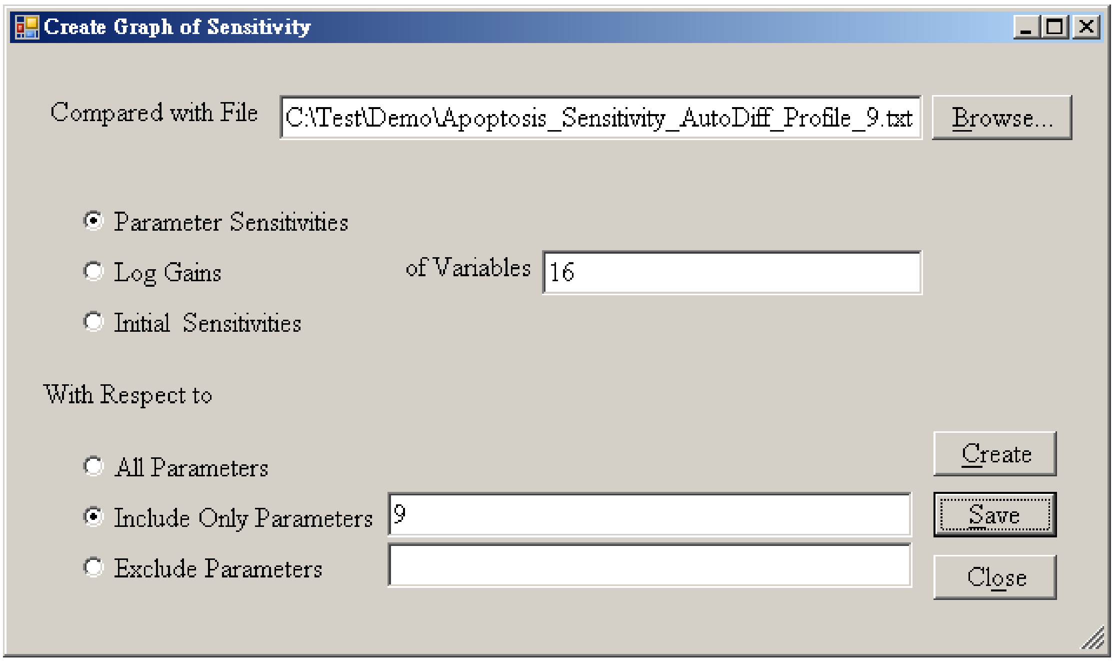

The output graph is shown in a pop-up window as follows:

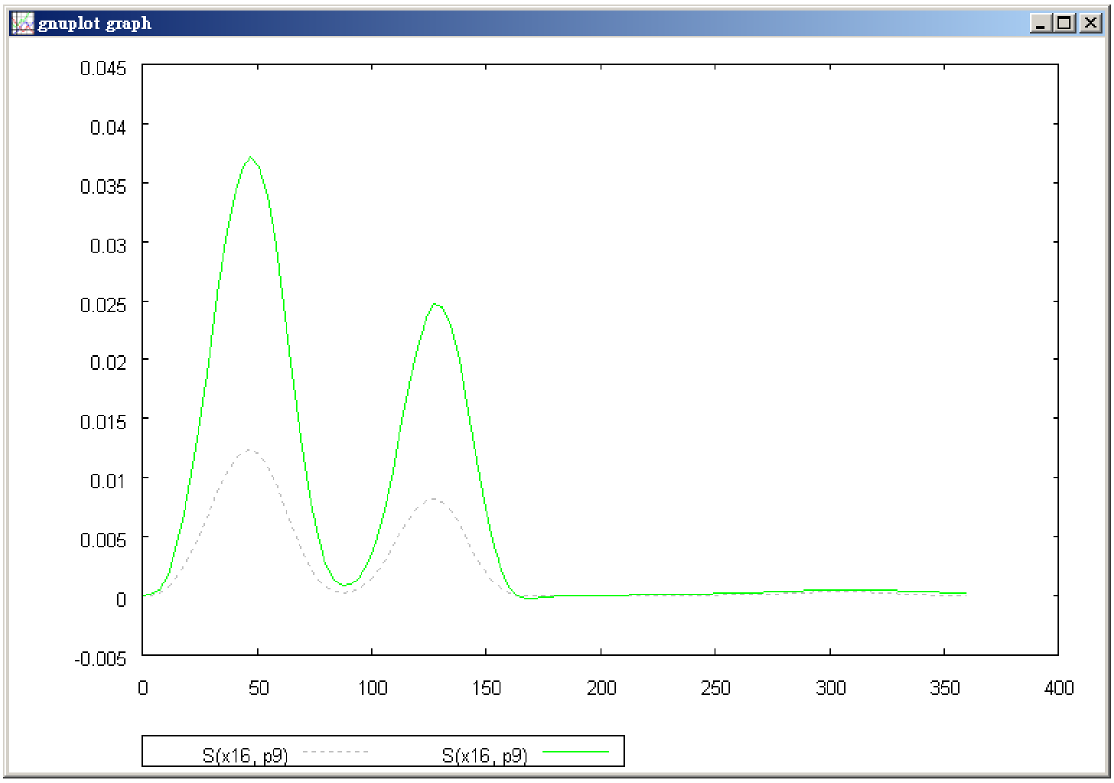

The gray dot line is the plot for the comparison data stored in the "Apoptosis_Sensitivity_AutoDiff_Profile_9.txt" file and the green line is the plot for the output data of current model.
